# Serum neuritin as a predictive biomarker of early neurological deterioration and poor prognosis after spontaneous intracerebral hemorrhage: a prospective cohort study

**DOI:** 10.3389/fneur.2024.1490023

**Published:** 2025-01-07

**Authors:** Yanwen Xu, Hanyu Zhu, Yuqi Su, Zhizhi Chen, Chuanliu Wang, Ming Yang, Feifei Jiang, Yunping Li, Yongming Xu

**Affiliations:** ^1^Department of Neurology, The Quzhou Affiliated Hospital of Wenzhou Medical University, Quzhou People’s Hospital, Quzhou, China; ^2^Operation Room, The Quzhou Affiliated Hospital of Wenzhou Medical University, Quzhou People’s Hospital, Quzhou, China; ^3^Department of Neurosurgery, The Quzhou Affiliated Hospital of Wenzhou Medical University, Quzhou People’s Hospital, Quzhou, China

**Keywords:** intracerebral hemorrhage, neuritin, early neurological deterioration, prognosis, severity, outcome

## Abstract

**Objective:**

Intracerebral hemorrhage (ICH) is a common cerebrovascular disease characterized by high mortality and disability rates. Neuritin, significantly expressed in injured brain tissues, is implicated in the molecular mechanisms underlying acute brain injury. We aimed to explore the prognostic and predictive value of serum neuritin in ICH.

**Methods:**

In this prospective cohort study, serum neuritin levels were measured at admission in 202 patients, on post-ICH days 1, 3, 5, 7, and 10 in 54 of these patients, and at the time of enrollment in 100 healthy controls. The Glasgow Coma Scale (GCS) and hematoma volume were used as severity indicators. A poor prognosis was defined as a modified Rankin Scale (mRS) score of 3–6 at 90 days after ICH. END was defined as a decrease of ≥2 points in the GCS score within 24 h of admission. A multivariate logistic regression model was used to assess the independent relationships between serum neuritin levels, END, and poor prognosis.

**Results:**

Serum neuritin levels were significantly increased at the time of patient admission, continued to rise on day 1, peaked on day 3, and then gradually diminished from day 5 until day 10. The levels remained substantially higher in patients compared to healthy controls throughout the 10-day period. The levels were independently related to GCS scores and hematoma volume. In subgroup analyses, the levels showed a linear relationship with the likelihood of experiencing END and poor prognosis at the 90-day mark after ICH. Additionally, the levels were independently associated with END, ordinal mRS scores, and poor prognosis. Under receiver operating characteristic (ROC) curve analysis, serum neuritin levels effectively predicted both END and poor prognosis. Two models incorporating GCS, hematoma volume, and serum neuritin levels were developed and represented using two nomograms separately to estimate END risks and poor prognosis. These models demonstrated clinical efficiency, stability, and validity in ROC, calibration, and decision curve analyses. Internal validation of the models was conducted using a randomly extracted subset of 101 patients. Furthermore, two specific weighted scoring systems were developed to optimize clinical prediction of poor prognosis and END after ICH.

**Conclusion:**

Elevated serum neuritin levels are strongly associated with disease severity, END, and 90-day poor neurological outcomes following ICH, establishing serum neuritin as a potential prognostic biomarker for ICH.

## 1 Introduction

Spontaneous intracerebral hemorrhage (sICH) pertains to the non-traumatic rupture of blood vessels resulting in the accumulation of blood within brain tissue, and it is correlated with high rates of disability and mortality (1, 2). Mechanisms underlying secondary brain injury after ICH are very complex, including inflammatory reactions, cellular apoptosis, perihematomal edema, and more, which are all related to poor outcomes for patients (3–5). In recent years, some biomarkers have been widely studied in ICH prognosis prediction and severity assessment (6, 7). Several factors, such as age, intraventricular hemorrhage, subtentorial hemorrhage, Glasgow Coma Scale (GCS), and hematoma volume, are associated with the prognosis of sICH (1, 8). Early neurological deterioration (END) is a common phenomenon, and its appearance is highly associated with poor prognosis in sICH patients (9, 10). Thus, it is significant to explore biomarkers of pertinence to END and poor prognosis following ICH.

Neuritin, also known as CPG15, is a newly described member of the family of neurotrophic factors (11). It possesses numerous properties, such as the facilitation of neuroplasticity, anti-inflammation, and anti-apoptosis (12). Neuritin expressions by animal neurons can be markedly up-regulated, and its supplementation significantly depressed neuroinflammation, reduced brain edema, and attenuated neuronal death in experimental ICH, ischemic stroke, subarachnoid hemorrhage, and Alzheimer’s disease (13–18). Thus, these features could identify neuritin as a potential endogenous mechanism for the minimization of secondary injury, which could imply its utility as a biomarker of brain injury. Here, we conducted an observational study to investigate the alteration of serum neuritin levels and its prognostic implications in ICH.

## 2 Materials and methods

### 2.1 Study design and participant selection

Consecutive recruitment of sICH patients admitted to Quzhou People’s Hospital was conducted from April 2020 to December 2022. The inclusion criteria were as follows: (1) first-ever stroke; (2) admission within 24 h of symptom onset; (3) age 18 years or older; (4) non-surgical treatment of hematoma; (5) intraparenchymal bleeding.

The exclusion criteria were the following: (1) hematoma caused by congenital or acquired coagulation abnormalities, hemorrhagic transformation of cerebral infarction, moyamoya disease, intracranial aneurysm, arteriovenous malformation, or neoplasms; (2) a history of other previous neurological diseases, such as ischemic or hemorrhagic stroke, intracranial tumors, or severe head trauma; and (3) specific diseases or conditions, such as infection in recent months, pregnancies, malignancies, uremia, liver cirrhosis, or chronic heart or lung disease (Supplementary Figure S1).

Additionally, healthy individuals who underwent physical examinations at our hospital were consecutively recruited as controls from May 2021 to July 2022. All controls met the following enrollment criteria: (1) no underlying diseases, including but not limited to hypertension, diabetes, hyperlipidemia, and coronary heart disease, and (2) normal results in routine blood tests, such as blood glucose, platelet count, hemoglobin concentration, and white blood cell count, in the normal ranges (Supplementary Figure S1).

This observational study was continually divided into a cross-sectional sub-study and a prospective cohort sub-study. In the cross-sectional assessment for investigating the longitudinal change of serum neuritin levels following ICH, venous blood was drawn at enrollment in controls, at admission in all patients, and at days 1, 3, 5, 7, and 10 after ICH in a portion of patients who were willing for blood collections at several time points. In the prospective cohort investigation for determining the feasibility of serum neuritin as a prognostic biomarker in ICH, all patients had their measurements of serum neuritin levels at admission and were observed for END and poor prognosis at the 90-day mark after ICH. This study was performed following principles set forth in the Declaration of Helsinki. The protocol of the current study was approved by the Institutional Review Committee at the Quzhou People’s Hospital (Opinion No: 2022–136). Written informed consent forms were obtained from patients’ relatives and controls themselves.

### 2.2 Acquired information

We collected basic information about the patients, including demographics (age and sex), vascular risk factors (hypertension, diabetes, and hyperlipidemia), medication history (use of statins, antiplatelet agents, and anticoagulants), and medical history. Stroke severity was assessed at admission using the GCS. All patients underwent baseline computed tomography scans of the head, and the hematoma volume was measured using the ABC/2 formula (19). Localizations of hematoma in lobular, subtentorial, intraventricular, or subarachnoid space were recorded. Upon arrival at the emergency center, non-invasive techniques were applied to reading systolic and diastolic blood pressures. A decrease of ≥2 points in GCS score within 24 h after admission was deemed as END (9). Patients with a modified Rankin Scale (mRS) score of ≥3 at post-stroke 90 days were considered to have a poor prognosis (20).

### 2.3 Immune analysis

Venous blood samples of controls were collected at their recruitment into this study. Peripheral blood specimens at admission were collected from all patients, and those at days 1, 3, 5, 7, and 10 after ICH were gained from some patients who consented to blood collections at multiple time points. Blood glucose, potassium, and C-reactive protein levels and white blood cell counts were measured by employing routine methods. A quick deposition of blood specimens into 5 mL gel-containing biochemistry tubes (Ningbo Siny Medical Technology Co., Ltd., China) was undertaken to measure serum neuritin levels. Once coagulation was observed, blood samples were spun at 2,000 *g* for 10 min. Subsequently, the supernatants were moved into Eppendorf tubes (Eppendorf Tubes^®^ BioBased, China) for prompt preservation at conditions of below 80°C for later analysis. Quantifications were conducted in batches of serum neuritin levels. In detail, a batch of serum samples, which were collected in a recent quarter, were thawed for measurements at the end of a quarter in order to ensure time from blood drawings to biomarker measurements did not exceed 3 months to prevent protein from breaking down. By applying a commercially available kit (Article Number: EH0944; Wuhan Fine Biotech Co., Ltd.), serum neuritin detection by enzyme-linked immunosorbent assay was in duplicate fulfilled in a blinded mode by the same proficient personnel in accordance with the manufacturer’s instructions. The sensitivity of the kit was 18.75 pg./mL, and the detection range was 31.25–2,000 pg./mL, with both intra-assay and inter-assay variabilities below 10%. The dual measurements were calculated to acquire the average values for the statistical assessments in the end. A high correlation existed between the double measurements (*ρ* = 0.998, *p* < 0.001; intraclass correlation coefficient = 0.998, *p* < 0.001). Using the Bland–Altman plot, a satisfactory consensus was ascertained between the two measurements (Supplementary Figure S2).

### 2.4 Statistical analysis

The SPSS 23.0 (SPSS Inc., Chicago, IL, United States), MedCalc 20 (MedCalc Software, Ltd., Ostend, Belgium), GraphPad Prism 7.01 (GraphPad Software Inc., San Diego, California, United States), and the software package R (version 3.5.1)^1^ were operated for plotting figures or statistical analyses. Normality evaluation by the Kolmogorov–Smirnov test was finished for measurement data, and subsequently, they were reported as means with standard deviations (SDs) or medians with 25–75 percentiles as deemed suitable. Qualitative data were presented as counts (proportions). The data comparisons between the two groups were completed using the chi-square test, Fisher exact test, *t*-test, or the Mann–Whitney *U* test as applicable. The Kruskal–Wallis *H* test was used to compare the differences in data among multiple groups. The Spearman correlation coefficient was generated to analyze the bivariate correlations. Subsequently, significantly correlated variables on univariate analysis (*p* < 0.05) were consolidated in the multifactorial linear regression model to identify variables that were independently relevant to serum neuritin levels. In order to discern independent predictors of poor prognosis indicated by mRS scores, mRS was regarded as an ordinal variable and a categorical variable (mRS 0–2 versus 3–6). The binary logistic regression model and ordinal regression model were constructed to ascertain independent predictors of poor prognosis. The variables with *p* < 0.05 on the univariate analysis should be integrated into the multivariable models for further analysis. Associations were expressed as odds ratios (ORs) and 95% confidence intervals (CIs). Similarly, independent predictors of END were determined using identical methods. Discrimination efficiency was assessed using receiver operating characteristic (ROC) curve analysis, with reporting of the area under the curve (AUC) and the corresponding 95% CI values. The restricted cubic splines were plotted to show a linear relationship, the combined models were visualized via the nomograms, calibration curves were generated to assess the stability of the models, and decision curves were produced to evaluate the clinical validity of the models. Half of all patients were randomly extracted for an internal validation of models. Weighted scoring systems were formed to optimize the prediction of poor prognosis and END after ICH. A two-sided *p*-value less than 0.05 signified a statistical difference.

## 3 Results

### 3.1 Participant selection and features

Initially, the consecutive recruitment of 269 patients with ICH was completed in compliance with the prespecified inclusion criteria in Supplementary Figure S1. In total, 67 patients were ineligible and removed from this study in accordance with the predefined exclusion requirements in Supplementary Figure S1. Finally, a collective of 202 ICH cases were retained for further analysis. The baseline characteristics of all 202 patients are listed in Supplementary Table S1. Moreover, among these 202 patients, mRS scores 0, 1, 2, 3, 4, 5, and 6 were found separately in 17, 21, 73, 32, 36, 17, and 6 patients (median, 2; 25th–75th percentiles, 2–4).

A total of 91 and 57 patients experienced adverse outcomes at 90 days and END post-ICH, respectively. Moreover, a total of 54 consented to blood collection at multiple time points. Supplementary Table S1 shows no significant differences in baseline characteristics between all patients and the 54 who agreed to blood collection at several time points post-ICH (all *p* > 0.05). In addition, 100 healthy controls were recruited. A total of 53 males and 47 females composed controls, with a mean age of 61.1 years (SD, 14.0 years), and controls encompassed 20 tobacco smokers and 19 alcohol consumers. Statistically, there were no significant differences in age, sex, smoking, and alcohol consumption between all patients and the controls (all *p* > 0.05).

### 3.2 Change of serum neuritin levels and its correlation with sickness severity

Serum neuritin levels of ICH patients promptly increased at admission, maintained elevated levels on day 1, reached the highest levels on day 3, and then gradually decreased from day 5 to day 10; and serum neuritin levels were significantly higher during the initial 10 days after ICH in patients than in controls (*p* < 0.001; Supplementary Figure S3). Serum neuritin levels in ICH patients were graphically shown to be significantly negatively correlated with GCS scores (*p* < 0.001; Supplementary Figure S4A) and to be substantially positively related to hematoma volume (*p* < 0.001; Supplementary Figure S4B). According to GCS, the patients were divided into three groups with scores of 3–8, 9–12, and 13–15 separately. In accordance with hematoma volume, the patients were categorized into two groups with bleeding sizes of below 30 mL and above 30 mL, respectively. In the form of a graph, serum neuritin levels were markedly highest in patients with the scores 3–8, were medium in those with the scores 9–12, and were profoundly lowest in those with the scores 13–15 (*p* < 0.001; Supplementary Figure S4C). In addition, serum neuritin levels were dramatically higher in patients with hematoma volume > 30 mL than in those with hematoma volume < 30 mL (*p* < 0.001; Supplementary Figure S4D). Moreover, within the framework of the Spearman test, serum neuritin levels were highly correlated with age, GCS scores, hematoma volume, diabetes mellitus, blood glucose levels, and intraventricular hemorrhage (all *p* < 0.05; Table 1). Moreover, a multivariate linear regression model was developed with the incorporation of the above-mentioned six significantly correlated variables, leading to the finding that admission serum neuritin levels were independently correlated with GCS scores and hematoma volume (both *p* < 0.05; Table 2).

**Table 1:** Factors correlated with serum neuritin levels after intracerebral hemorrhage.

	ρ	*P-*value
Gender (male/female)	−0.026	0.710
Age (y)	0.143	**0.042**
Current cigarette smoking	−0.044	0.533
Alcohol abuse	0.017	0.812
Hypertension	−0.066	0.349
Diabetes mellitus	0.149	**0.034**
Hyperlipidemia	0.114	0.105
Use of statins drugs	−0.013	0.856
Use of antiplatelet drugs	−0.004	0.959
Use of anticoagulation drugs	−0.002	0.973
Hospital admission time (h)	−0.080	0.257
Blood-sampling time (h)	−0.108	0.126
Lobar hemorrhage	−0.042	0.551
Infratentorial hemorrhage	−0.013	0.852
Intraventricular hemorrhage	0.231	**0.001**
Subarachnoid hemorrhage	0.058	0.413
GCS scores	−0.344	**<0.001**
Hematoma volume (ml)	0.436	**<0.001**
Systolic arterial pressure (mmHg)	0.021	0.768
Diastolic arterial pressure (mmHg)	−0.033	0.643
Blood glucose levels (mmol/l)	0.149	**0.035**
Blood potassium levels (mmol/l)	0.076	0.280
Blood leucocyte count (×10^9^/l)	0.028	0.696
Blood C-reactive protein levels (mg/l)	0.085	0.229

Correlations were analyzed using Spearman test. GCS indicates Glasgow coma scale. Bold values indicate *p* < 0.05.

**Table 2:** Multivariate linear regression analysis between elevated serum neuritin levels and other variables.

	*β* (95% confidence interval)	*P-*value
Age (y)	0.058 (−0.354 to 0.470)	0.783
Diabetes mellitus	8.457 (−5.422 to 22.337)	0.231
Intraventricular hemorrhage	15.274 (−0.612 to 31.160)	0.059
GCS scores	−2.697 (−5.357 to −0.037)	**0.047**
Hematoma volume (ml)	1.290 (0.772 to 1.808)	**0.001**
Blood glucose levels (mmol/l)	0.354 (−1.566 to 2.275)	0.716

Correlations were yielded using the multivariate linear regression analysis. Bold values indicate *p* < 0.05.

### 3.3 Relationship between serum neuritin levels and post-ICH neurological outcome

Serum neuritin levels were significantly increased in patients with poor prognoses, as opposed to those with good prognoses (*p* < 0.001; Supplementary Figure S5).

**Figure 1:**
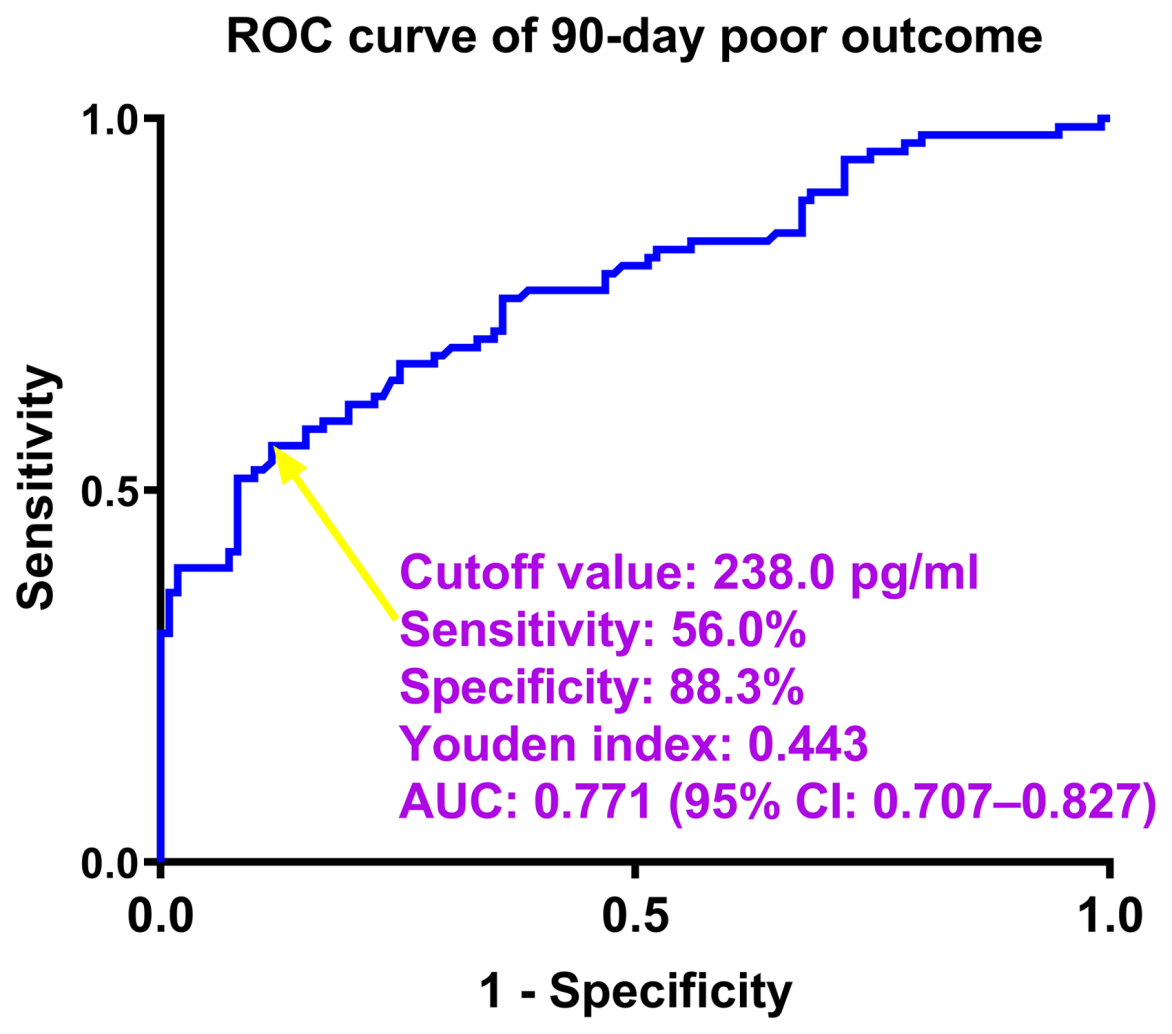
Predictive ability of serum neuritin levels for poor outcomes at 90 days after intracerebral hemorrhage under the receiver-operating characteristic curve. Serum neuritin levels significantly predicted post-stroke 90-day poor outcome (area under the curve, 0.771; 95% confidence interval, 0.707–0.827). Serum neuritin levels >238.0 pg./mL distinguished patients with the development of poor 90-day outcomes with specificity and sensitivity values of 88.3 and 56.0% (maximum Youden index J, 0.4433), respectively. ROC denotes receiver operating characteristic; AUC, area under the curve; 95% CI, 95% confidence interval. The yellow arrow points to the cutoff value of serum neuritin levels.

Using the as a continuous variable in a linear analysis, it showed a clear positive correlation with the serum neuritin levels of patients (*p* < 0.001; Supplementary Figure S6A). Furthermore, the ICH patients were classified into seven groups based on their mRS scores from 0 to 6. There was a marked trend by a graphical representation that serum neuritin levels were increased in the order of the scores from 0 to 6 (*p* < 0.001; Supplementary Figure S6B). In the context of restricted cubic spline assessment, there was a linear concentration-response relationship between serum neuritin and risk of 90-day poor outcome (*P* for non-linear >0.05; Supplementary Figure S7). Moreover, under the ROC curve, serum neuritin levels efficaciously predicted post-stroke 90-day poor outcomes among this group of ICH patients (AUC, 0.771; 95% CI, 0.707–0.827), and serum neuritin levels >238.0 pg./mL distinguished patients with development of poor 90-day outcomes with specificity and sensitivity values of 88.29 and 56.04% (Youden index J, 0.4433), respectively (Figure 1).

From the perspective of statistics, mRS was recognized as an ordinal variable. Serum neuritin levels, GCS scores, hematoma volume, diabetes mellitus, blood C-reactive protein levels, and intraventricular hemorrhage significantly differed among seven groups, which were divided at a base of mRS (all *p* < 0.05; Table 3). With the help of the multifactorial ordinal regression analysis, GCS scores, hematoma volume, and serum neuritin levels were independently associated with mRS scores at 90 days after ICH (all *p* < 0.01; Table 4).

**Table 3:** Factors associated with ordinal 90-day modified Rankin scale scores after intracerebral hemorrhage.

	Ninety-day ordinal modified Rankin scale score							
	0	1	2	3	4	5	6	*P-*value
Gender (male/female)	14/3	12/9	45/28	13/19	24/12	8/9	4/2	0.091
Age (y)	61 (48–67)	63 (45–70)	62 (52–70)	64 (52–72)	65 (53–76)	66 (65–77)	80 (69–86)	0.143
Current cigarette smoking	4 (23.5%)	5 (23.8%)	18 (24.7%)	10 (31.3%)	4 (11.1%)	4 (23.5%)	3 (50.0%)	0.355
Alcohol abuse	5 (29.4%)	5 (23.8%)	17 (23.3%)	10 (31.3%)	10 (27.8%)	5 (29.4%)	3 (50.0%)	0.874
Hypertension	12 (70.6%)	14 (66.7%)	44 (60.3%)	19 (59.4%)	25 (69.4%)	10 (58.8%)	5 (83.3%)	0.823
Diabetes mellitus	1 (5.9%)	5 (23.8%)	11 (15.1%)	5 (15.6%)	11 (30.6%)	8 (47.1%)	1 (16.7%)	**0.043**
Hyperlipidemia	2 (11.8%)	2 (9.5%)	14 (19.2%)	6 (18.8%)	6 (16.7%)	6 (35.3%)	3 (50.0%)	0.268
Use of statins drugs	1 (5.9%)	1 (4.8%)	9 (12.3%)	4 (12.5%)	5 (13.9%)	3 (17.7%)	2 (33.3%)	0.614
Use of antiplatelet drugs	0 (0.0%)	3 (14.3%)	11 (15.1%)	7 (21.9%)	6 (16.7%)	2 (11.8%)	0 (0.0%)	0.191
Use of anticoagulation drugs	0 (0.0%)	1 (4.8%)	6 (8.2%)	4 (12.5%)	2 (5.6%)	2 (11.8%)	0 (0.0%)	0.485
Hospital admission time (h)	6.0 (4.0–7.4)	5.0 (4.0–7.0)	6.0 (4.0–10.0)	6.0 (4.6–7.8)	5.0 (3.4–7.0)	5.0 (3.0–8.0)	4.0 (2.5–6.0)	0.321
Blood-sampling time (h)	7.0 (5.0–8.0)	5.9 (4.5–8.0)	7.0 (5.0–11.3)	6.8 (5.0–9.0)	6.0 (4.3–8.3)	5.0 (4.0–9.0)	4.8 (3.0–7.0)	0.421
Lobar hemorrhage	3 (17.7%)	4 (19.1%)	12 (16.4%)	12 (37.5%)	6 (16.7%)	6 (35.3%)	2 (33.3%)	0.220
Infratentorial hemorrhage	5 (29.4%)	2 (9.5%)	11 (15.1%)	3 (9.4%)	4 (11.1%)	3 (17.7%)	1 (16.7%)	0.646
Intraventricular hemorrhage	1 (5.9%)	1 (4.8%)	9 (12.3%)	4 (12.5%)	5 (13.9%)	9 (52.9%)	1 (16.7%)	**0.008**
Subarachnoid hemorrhage	0 (0.0%)	3 (14.3%)	2 (2.7%)	2 (6.3%)	4 (11.1%)	2 (11.8%)	0 (0.0%)	0.203
GCS scores	14 (13–14)	13 (13–14)	13 (12–14)	11 (10–13)	11 (9–12)	9 (7–10)	10 (4–11)	**<0.001**
Hematoma volume (ml)	4.2 (3.2–6.7)	11.7 (4.9–14.9)	13.2 (7.9–19.5)	15.7 (11.1–23.6)	23.8 (12.8–34.5)	38.3 (30.1–50.0)	42.9 (31.9–52.7)	**<0.001**
Systolic AP (mmHg)	157 (130–177)	154 (145–165)	157 (140–169)	157 (141–165)	155 (141–175)	151 (134–164)	162 (144–170)	0.992
Diastolic AP (mmHg)	88 (81–95)	87 (80–98)	90 (76–99)	89 (76–101)	94 (83–102)	87 (78–98)	91 (82–107)	0.787
Blood glucose levels (mmol/l)	5.8 (5.0–7.7)	8.0 (5.5–9.3)	6.6 (5.0–8.2)	6.2 (5.4–8.2)	7.7 (5.7–9.5)	8.3 (6.2–11.0)	8.8 (6.2–16.5)	0.063
Blood potassium levels (mmol/l)	3.8 (3.5–4.0)	3.6 (3.5–3.8)	3.8 (3.5–3.9)	3.6 (3.4–3.8)	3.7 (3.6–4.0)	3.8 (3.7–4.0)	3.5 (3.4–3.7)	0.246
Blood leucocyte count (×10^9^/l)	6.7 (5.9–8.5)	7.5 (6.2–10.6)	8.5 (6.5–9.6)	6.9 (5.4–9.7)	6.9 (5.6–10.3)	7.7 (6.0–12.5)	7.7 (7.3–8.2)	0.630
Blood CRP levels (mg/l)	4.8 (4.0–11.4)	6.4 (4.0–14.0)	11.2 (5.8–15.2)	11.5 (6.0–15.0)	13.7 (10.0–25.6)	10.0 (8.0–15.8)	10.0 (4.0–15.6)	**0.041**
Blood neuritin levels (pg/ml)	181.1 (134.8–201.2)	186.1 (170.3–200.8)	194.8 (169.3–226.1)	214.4 (188.5–239.0)	247.1 (203.0–239.0)	263.6 (242.9–283.7)	259.1 (252.5–271.7)	**<0.001**

Data were shown as median (25th–75th percentiles) or counts (percentages) where appropriate. Statistical methods for intergroup comparison included the Mann–Whitney *U* test, Pearson chi-square test or Fisher’s exact test as appropriate. GCS denotes Glasgow coma scale; CRP, C-reactive protein; AP, arterial pressure. Bold values indicate *p* < 0.05.

**Table 4:** Multifactorial ordinal regression analysis of risk factors for modified Rankin scale scores at 90 days after intracerebral hemorrhage.

	Odds ratio (95% confidence interval)	*P-*value
Diabetes mellitus	1.692 (0.878–3.261)	0.116
Intraventricular hemorrhage	1.160 (0.542–2.479)	0.704
GCS scores	0.771 (0.676–0.880)	**0.001**
Hematoma volume (ml)	1.105 (1.073–1.138)	**0.001**
Blood C-reactive protein levels (mg/l)	1.015 (0.991–1.040)	0.231
Blood neuritin levels (pg/ml)	1.013 (1.006–1.019)	**0.001**

Results were showed as odds ratio (95% confidence interval) according to the multifactorial ordinal regression analysis. Bold values indicate *p* < 0.05.

As shown in Table 5, patients with a poor prognosis, relative to those presenting with a good prognosis, tended to have significantly higher age, hematoma volume, blood glucose levels, and serum neuritin levels, were likely to exhibit substantially lower GCS scores, and were prone to display markedly higher percentages of diabetes mellitus and intraventricular hemorrhage (all *p* < 0.05). By applying the binary logistic regression analysis, GCS scores, hematoma volume, and serum neuritin levels emerged as the three independent predictors of poor prognosis at 90 days after ICH (all *p* < 0.01; Table 6).

**Table 5:** Factors associated with 90-day poor outcome after intracerebral hemorrhage.

	Poor outcome	Good outcome	*P-*value
Gender (male/female)	49/42	71/40	0.145
Age (y)	65.2 ± 13.6	60.9 ± 13.8	**0.026**
Current cigarette smoking	21 (23.1%)	27 (24.3%)	0.836
Alcohol abuse	28 (30.8%)	27 (24.3%)	0.306
Hypertension	59 (64.8%)	70 (63.1%)	0.794
Diabetes mellitus	25 (27.5%)	17 (15.3%)	**0.034**
Hyperlipidemia	21 (23.1%)	18 (16.2%)	0.219
Use of statins drugs	14 (15.4%)	11 (9.9%)	0.240
Use of antiplatelet drugs	15 (16.5%)	14 (12.6%)	0.435
Use of anticoagulation drugs	8 (8.8%)	7 (6.3%)	0.503
Hospital admission time (h)	5.0 (3.6–7.0)	6.0 (4.0–9.0)	0.136
Blood-sampling time (h)	6.0 (4.5–8.5)	7.0 (4.9–9.8)	0.170
Lobar hemorrhage	26 (28.6%)	19 (17.1%)	0.052
Infratentorial hemorrhage	11 (12.1%)	18 (16.2%)	0.405
Intraventricular hemorrhage	19 (20.9%)	11 (9.9%)	**0.029**
Subarachnoid hemorrhage	8 (8.8%)	5 (4.5%)	0.217
GCS scores	11 (9–12)	13 (12–14)	**<0.001**
Hematoma volume (ml)	25.5 (13.8–34.5)	11.5 (6.4–18.3)	**<0.001**
Systolic arterial pressure (mmHg)	154.2 ± 22.9	154.0 ± 21.0	0.937
Diastolic arterial pressure (mmHg)	90.8 ± 16.4	88.7 ± 14.3	0.329
Blood glucose levels (mmol/l)	7.4 (5.7–9.5)	6.6 (5.1–8.3)	**0.047**
Blood potassium levels (mmol/l)	3.7 (3.5–3.9)	3.7 (3.5–4.0)	0.658
Blood leucocyte count (×10^9^/l)	7.1 (5.6–9.8)	7.8 (6.1–9.6)	0.422
Blood C-reactive protein levels (mg/l)	12.0 (7.0–16.1)	10.0 (4.7–14.1)	0.070
Blood neuritin levels (pg/ml)	242.7 (207.7–261.7)	191.5 (163.7–221.3)	**<0.001**

Data were shown as mean ± standard deviation, median (25th–75th percentiles) or count (percentage) where appropriate. Statistical methods for intergroup comparison included the *t-*test, Mann–Whitney *U* test, Pearson chi-square test or Fisher’s exact test as appropriate. GCS denotes Glasgow coma scale. Bold values indicate *p* < 0.05.

**Table 6:** Multivariate logistic regression analysis for risk factors of poor outcome in intracerebral hemorrhage.

	Odds Ratio (95% Confidence Interval)	*P-*value
Age (y)	1.008 (0.980–1.038)	0.573
Diabetes mellitus	1.809 (0.705–4.641)	0.218
Intraventricular hemorrhage	1.175 (0.382–3.615)	0.779
GCS scores	0.689 (0.562–0.845)	**0.001**
Hematoma volume (ml)	1.079 (1.032–1.127)	**0.001**
Blood glucose levels (mmol/l)	1.039 (0.905–1.193)	0.588
Blood neuritin levels (pg/ml)	1.018 (1.008–1.028)	**0.001**

Results were showed as odds ratio (95% confidence interval) according to the binary logistic regression analysis. Bold values indicate *p* < 0.05.

**Figure 2:**
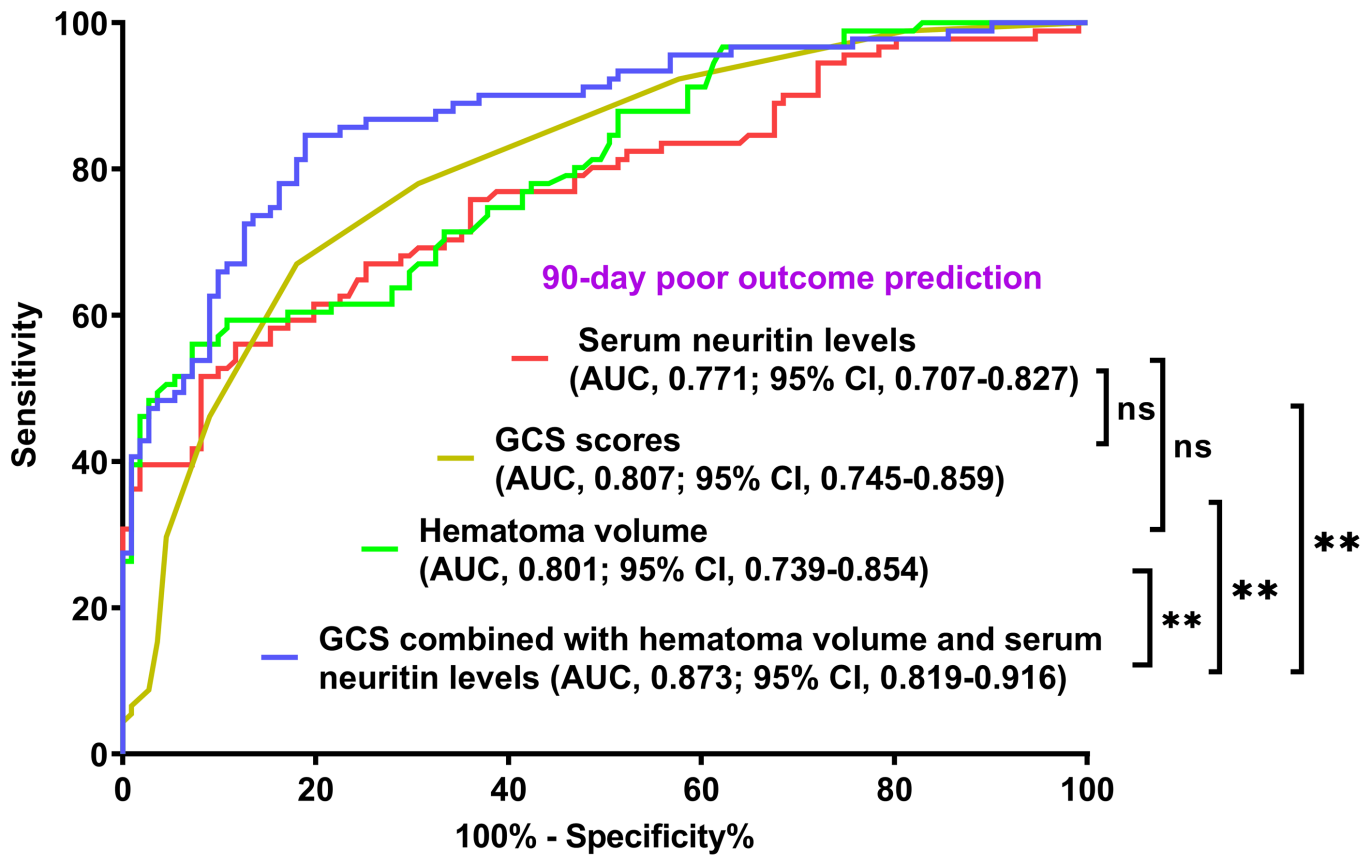
Comparison of discriminatory capability with respect to serum neuritin levels, Glasgow coma scale scores, hematoma volume, and combined model for 90-day poor outcome following intracerebral hemorrhage under the receiver operating characteristic curve. Prognostic predictive ability of serum neuritin level (area under curve, 0.771; 95% confidence interval, 0.707–0.827) was similar to those of Glasgow coma scale scores (area under curve, 0.807; 95% confidence interval, 0.745–0.859; *p* = 0.408) and hematoma volume (area under curve, 0.801; 95% confidence interval, 0.739–0.854; *p* = 0.451). Combined models, which contained Glasgow coma scale scores, hematoma volume, and serum neuritin levels, represented higher prognostic predictive efficiency than any of them (all ^**^p < 0.01). GCS means Glasgow coma scale; AUC, area under the curve; 95% CI, 95% confidence interval; ns, non-significant.

**Figure 3:**
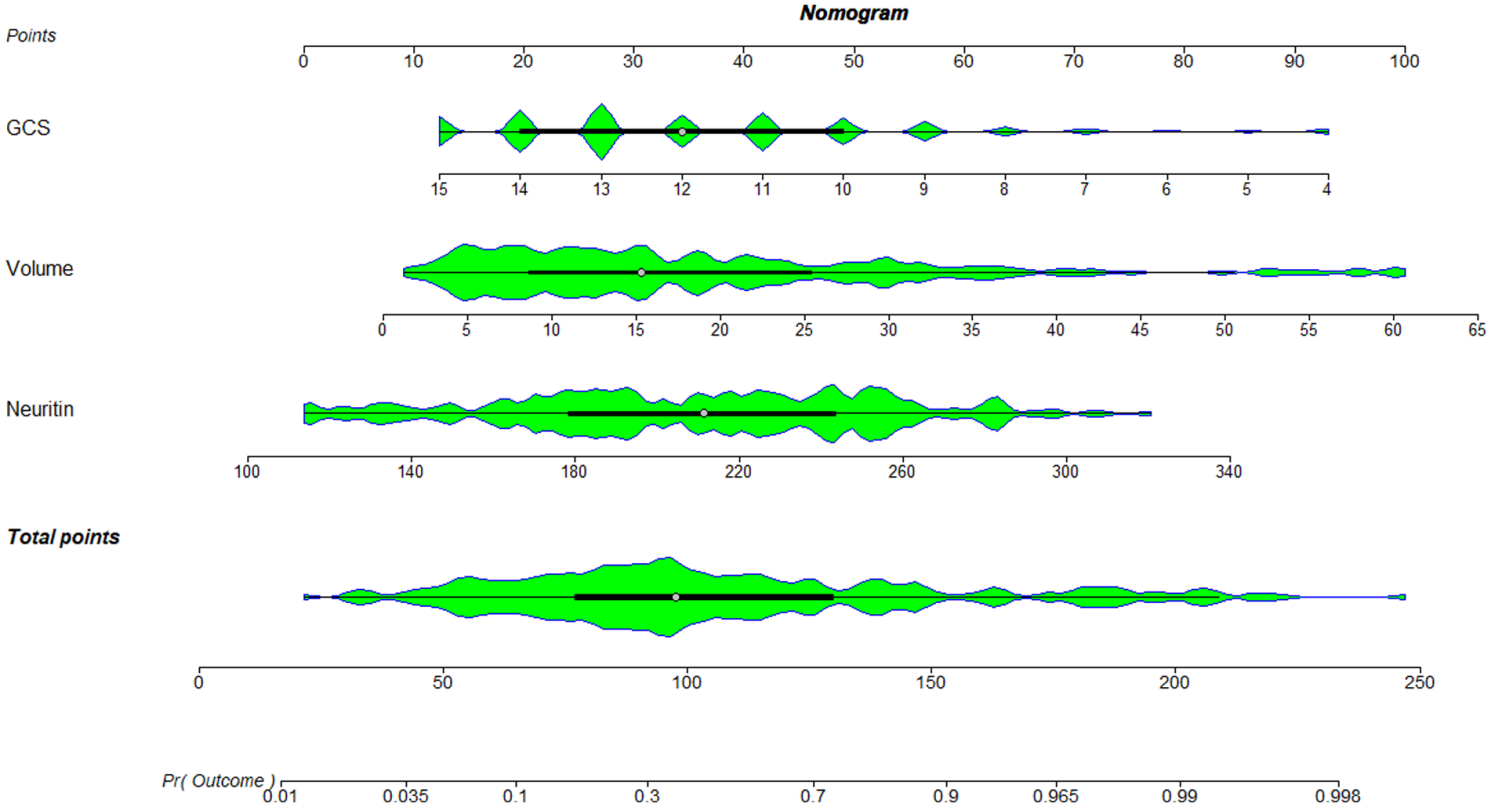
Nomogram combining serum neuritin levels at admission, Glasgow coma scale scores, and hematoma volume for predicting post-stroke 90-day poor prognosis. A nomogram was established to assess how the combination model was used to predict a poor prognosis at 90 days after intracerebral hemorrhage. GCS denotes the Glasgow coma scale, which is volume and hematoma volume.

**Figure 4:**
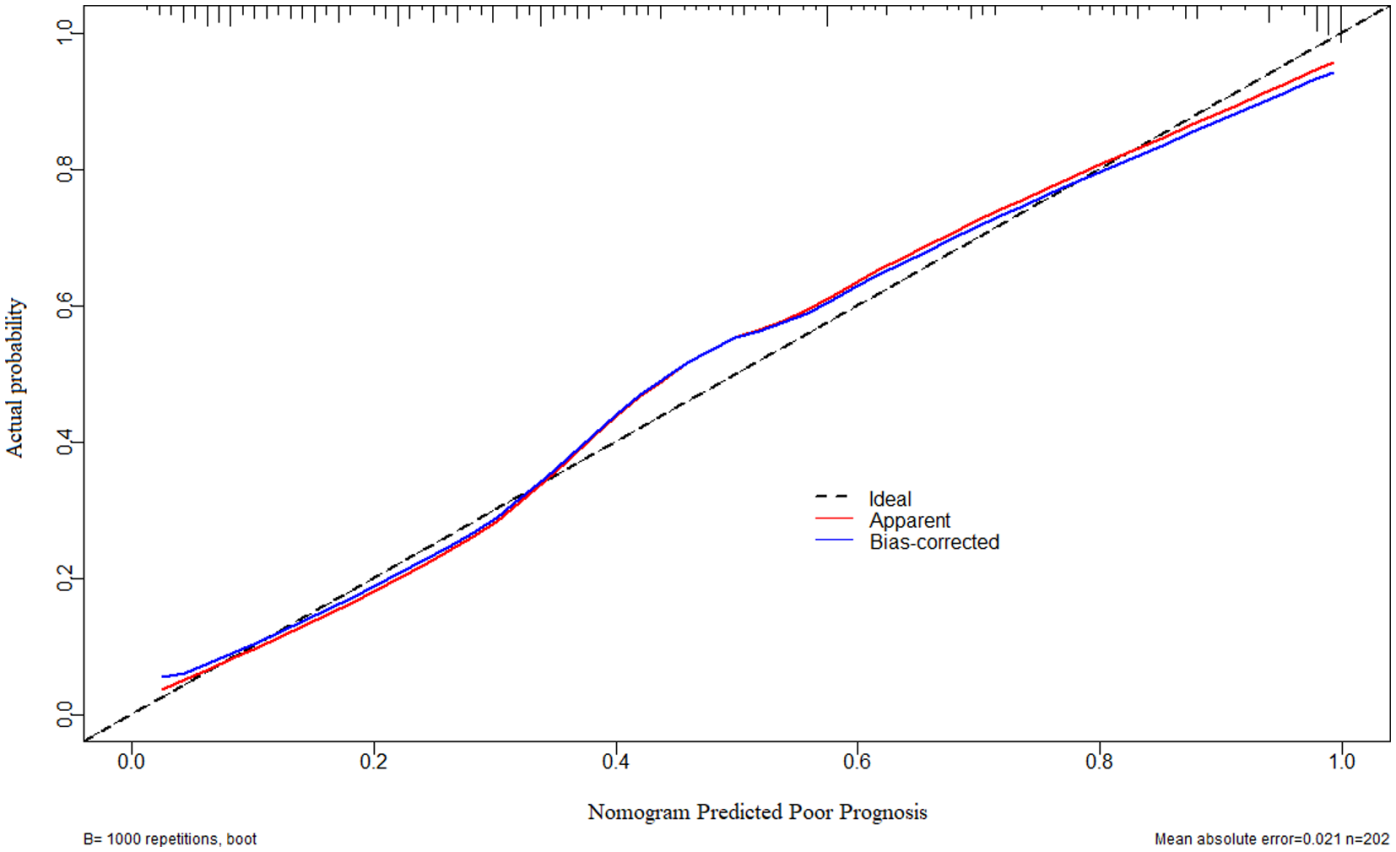
Calibration curve verifying the stability of a prognostic prediction model containing serum neuritin levels at admission, Glasgow coma scale scores, and hematoma volume after intracerebral hemorrhage. Under the calibration curve, such a prediction model was relatively stable.

**Figure 5:**
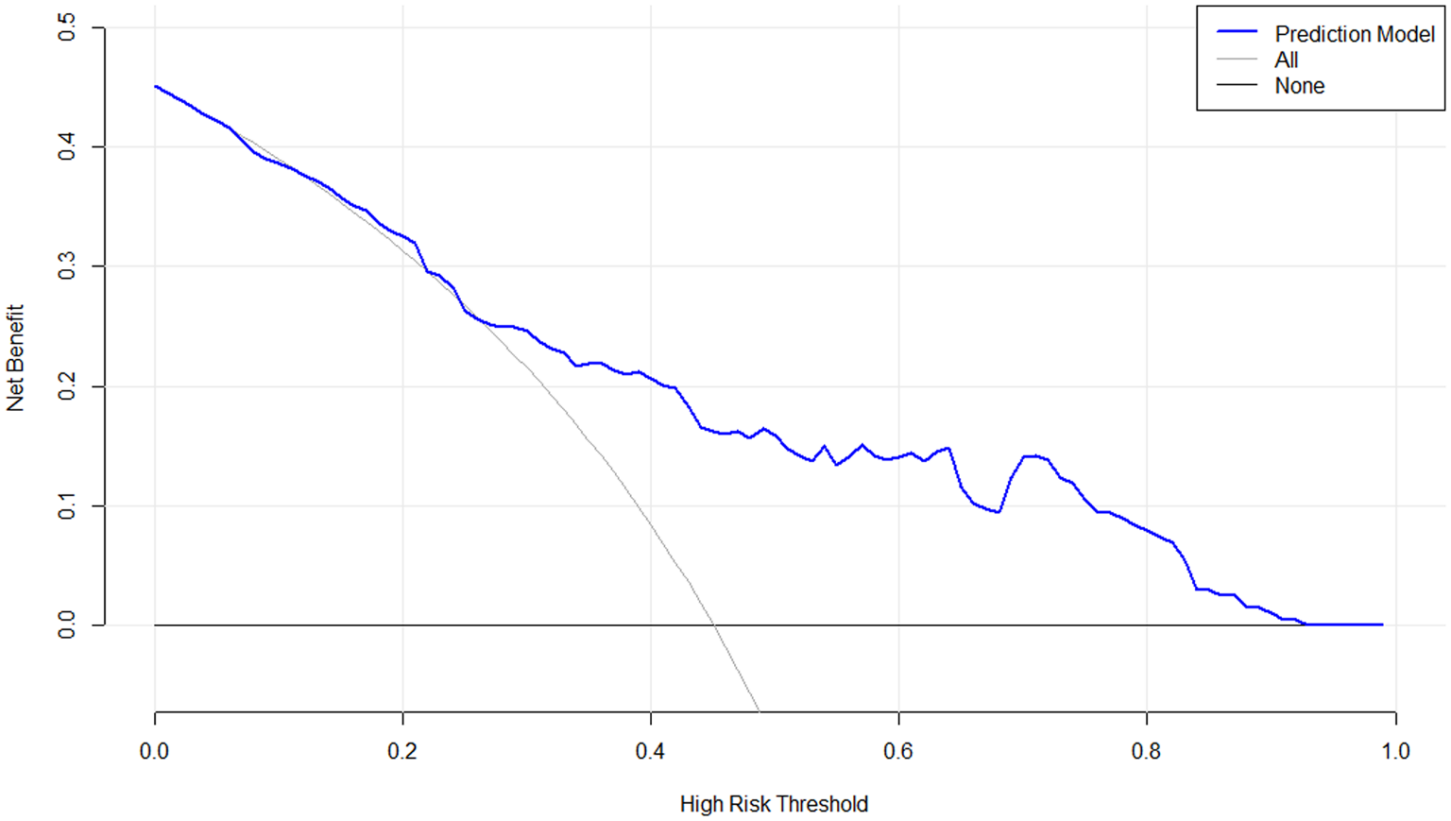
Decision curve confirming the clinical benefit of a prognostic prediction model containing serum neuritin levels at admission among patients with intracerebral hemorrhage. Decision curve analysis demonstrated that such a prediction model combining serum neuritin levels at admission, Glasgow coma scale scores, and hematoma volume, relatively speaking, had a high clinical benefit.

As displayed in Figure 2, the ability of serum neuritin levels in prognosticating poor prognosis was equivalent to those of GCS scores (AUC, 0.807; 95% CI, 0.745–0.859; *p* = 0.408) and hematoma volume (AUC, 0.801; 95% CI, 0.739–0.854; *p* = 0.451). The independent predictors of poor prognosis, i.e., GCS, hematoma volume, and serum neuritin, were combined to configure a model, which was delineated by adopting a nomogram for visual representation (Figure 3). The combined model represented higher prognostic and predictive efficiency than any of GCS, hematoma volume, and serum neuritin (all *p* < 0.01; Figure 2). Under the calibration curve, the prediction model ran acceptably stably (Figure 4). Using decision curve analysis, the prediction model had satisfactory clinical fitness (Figure 5). Furthermore, 101 patients were randomly extracted from the whole group of 202 patients. Supplementary Table S1 showed no significant distinctions of baseline features between the whole group and the extracted 101 patients (all *p* > 0.05). There were no substantial differences in terms of the predictive ability of both serum neuritin levels and the combination model in anticipating poor prognosis under the ROC curve between all 202 patients and the extracted 101 patients (both *p* > 0.05; Supplementary Figures S8, S9). Moreover, we defined GCS scores of 3–8 as 2, 9–12 as 1, and 13–15 as 0. Hematoma volume ≥ 30 mL was defined as 1, and hematoma volume < 30 mL was defined as 0. Serum neuritin levels ≥238.0 pg./mL were defined as 1, while levels less than 238.0 pg./mL were defined as 0. Percentages of patients with poor prognosis, which were 13.0% (10/77), 44.6% (29/65), 78.1% (25/32), 94.7% (18/19), and 100% (9/9) successively, were gradually increased in the order of the total scores from 0 to 4.

### 3.4 Relationship between serum neuritin levels and END after ICH

**Figure 6:**
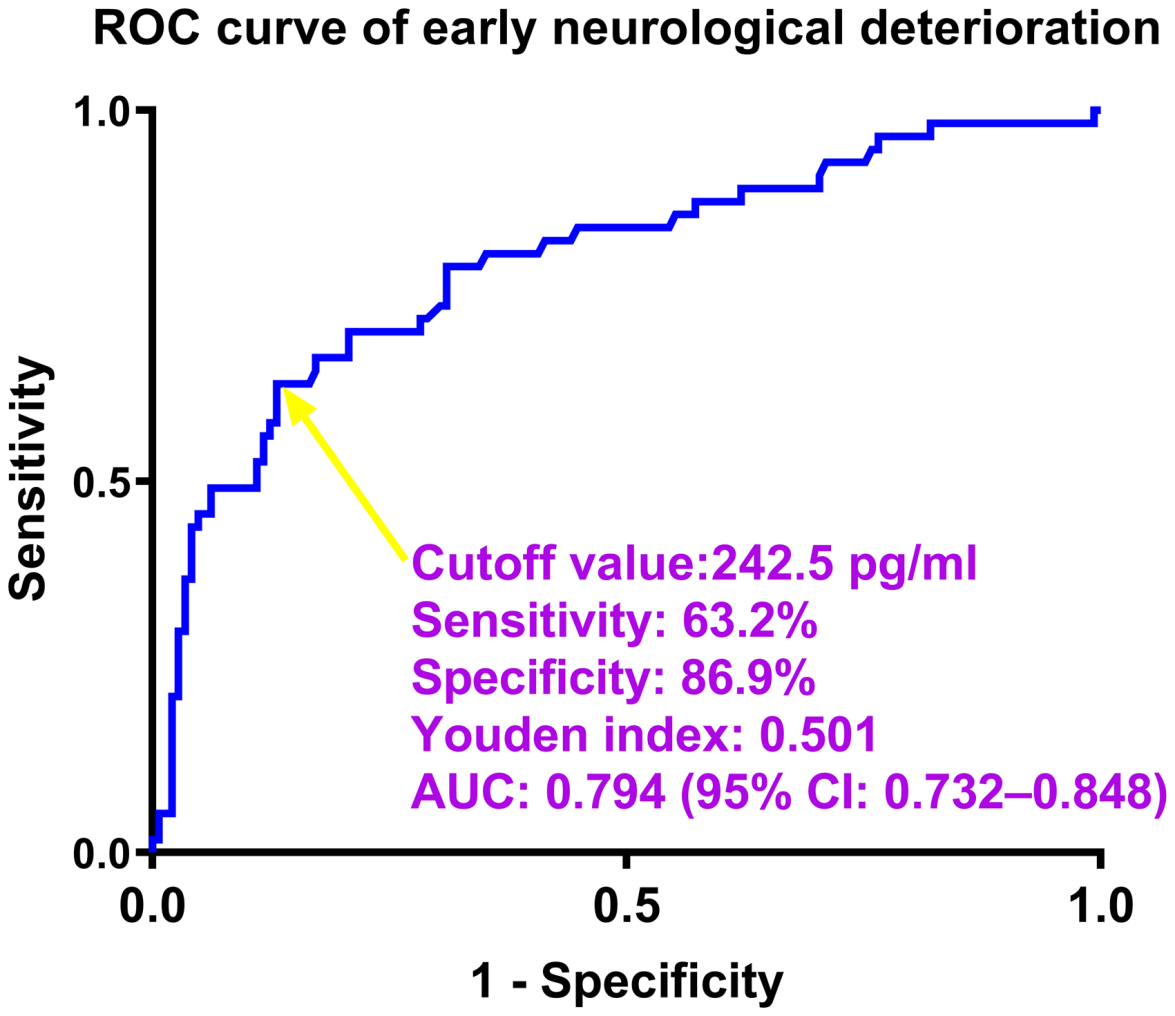
Predictive ability of serum neuritin levels for early neurological deterioration after intracerebral hemorrhage under the receiver-operating characteristic curve. Serum neuritin levels significantly predicted early neurological deterioration (area under the curve, 0.794; 95% confidence interval, 0.732–0.848). Serum neuritin levels >242.5 pg./mL distinguished patients with the development of early neurological deterioration with specificity and sensitivity values of 86.9 and 63.2% (maximum Youden index J, 0.501), respectively. ROC means receiver operating characteristic; AUC, area under the curve; 95% CI, 95% confidence interval. A yellow arrow points to the cutoff value of serum neuritin levels.

Serum neuritin levels in patients suffering from END were significantly higher than those in those without (*p* < 0.001; Supplementary Figure S10). By utilizing a restricted cubic spline, serum neuritin levels were linearly correlated with END probability (*P* for non-linear >0.05; Supplementary Figure S11). Moreover, under the ROC curve, serum neuritin levels efficiently predicted END among this group of ICH patients (AUC, 0.794; 95% CI, 0.732–0.848), and serum neuritin levels >242.5 pg./mL distinguished patients at the development of END with specificity and sensitivity values of 86.90 and 63.16% (Youden index J, 0.5005), respectively (Figure 6).

**Figure 7:**
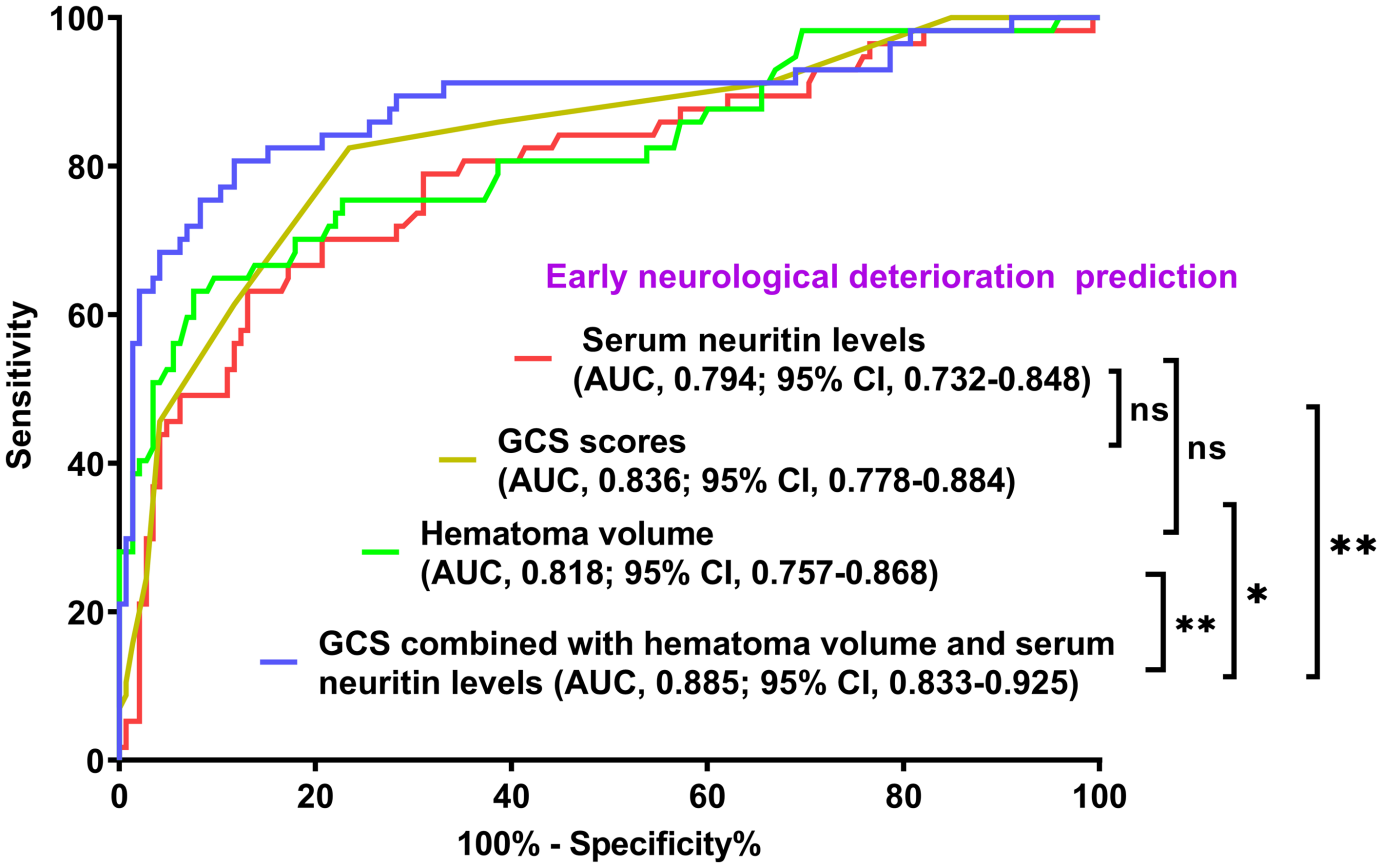
Comparison of discriminatory capability with respect to serum neuritin levels, Glasgow Coma Scale scores, and hematoma volume for early neurological deterioration following intracerebral hemorrhage under the receiver operating characteristic curve. The predictive ability of serum neuritin level (area under the curve, 0.794; 95% confidence interval, 0.732–0.848) was similar to those of Glasgow coma scale scores (area under the curve, 0.836; 95% confidence interval, 0.778–0.884; *p* = 0.314) and hematoma volume (area under the curve, 0.818; 95% confidence interval, 0.757–0.868; *p* = 0.543). Combined models containing the Glasgow Coma Scale scores, hematoma volume, and serum neuritin levels represented higher early neurological deterioration predictive efficiency than any of them (^*^p < 0.05 or ^**^p < 0.01). GCS means Glasgow coma scale; AUC, area under the curve; 95% CI, 95% confidence interval; ns, non-significant.

**Figure 8:**
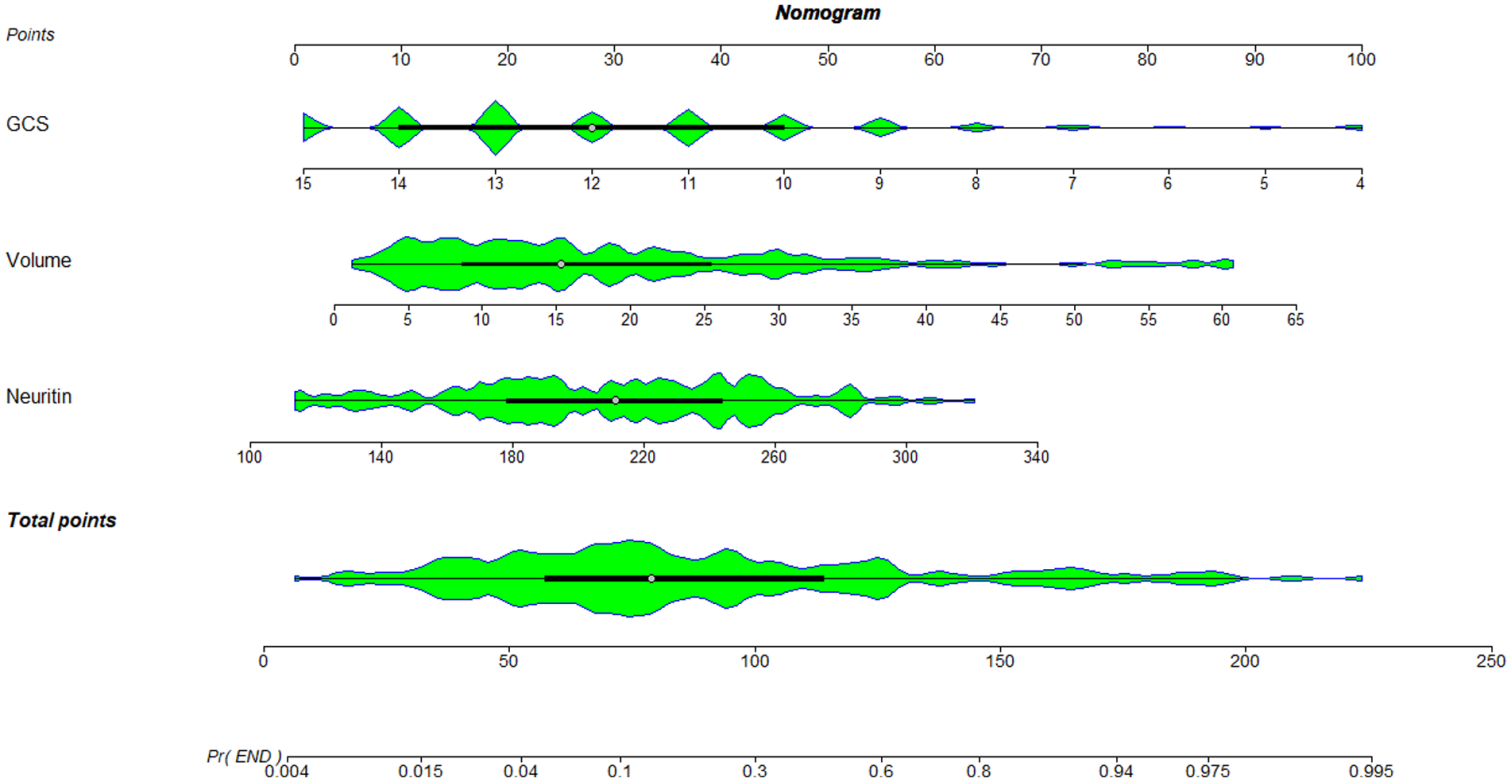
Nomogram combining serum neuritin levels at admission, Glasgow coma scale scores, and hematoma volume for predicting early neurological deterioration. A nomogram was established to assess how the combination model was used to predict early neurological deterioration after intracerebral hemorrhage. GCS denotes the Glasgow coma scale, which is volume and hematoma volume.

**Figure 9:**
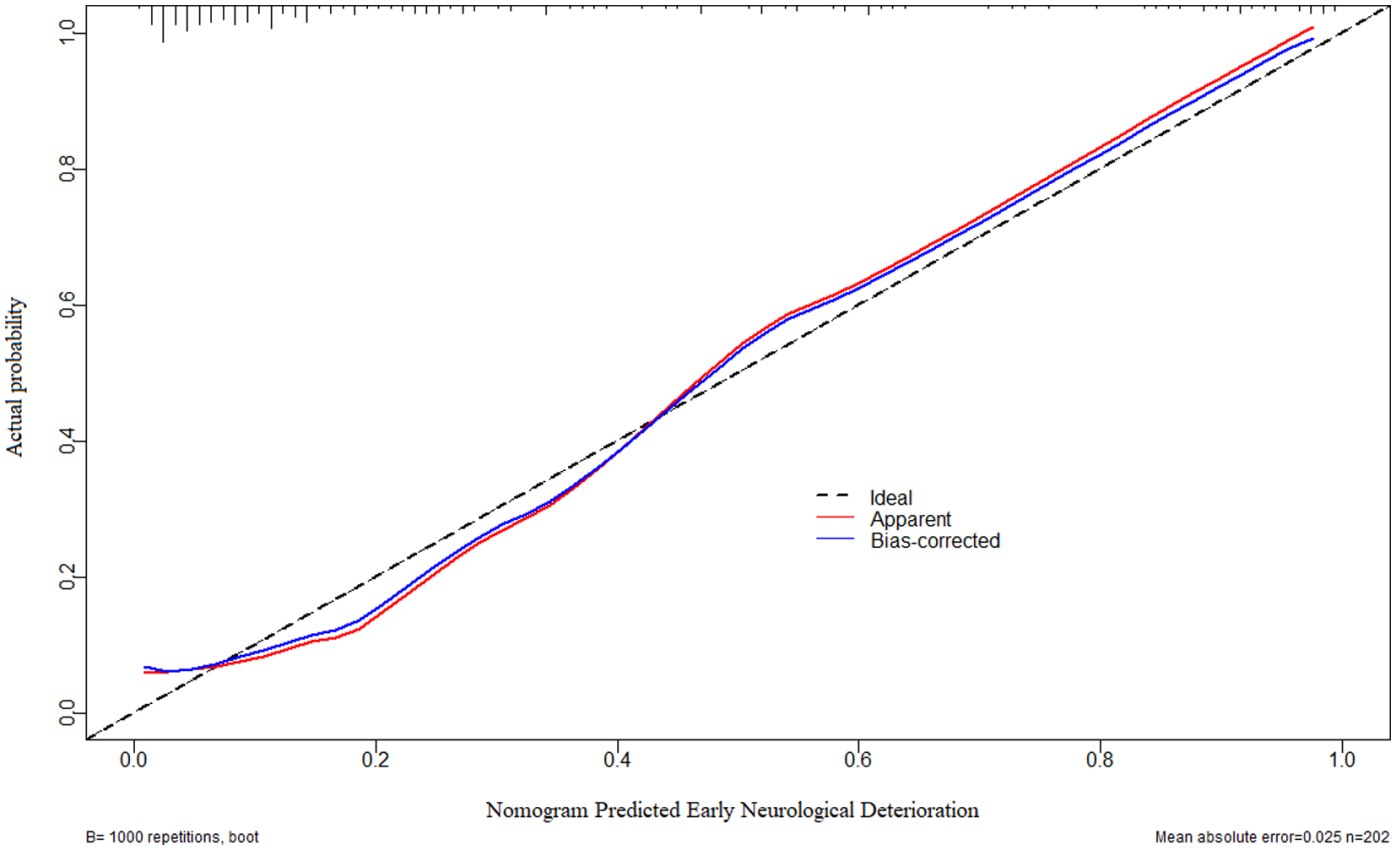
Calibration curve verifying the stability of early neurological deterioration prediction model containing serum neuritin levels at admission, Glasgow coma scale scores, and hematoma volume after intracerebral hemorrhage. Under the calibration curve, such a prediction model was relatively stable.

**Figure 10:**
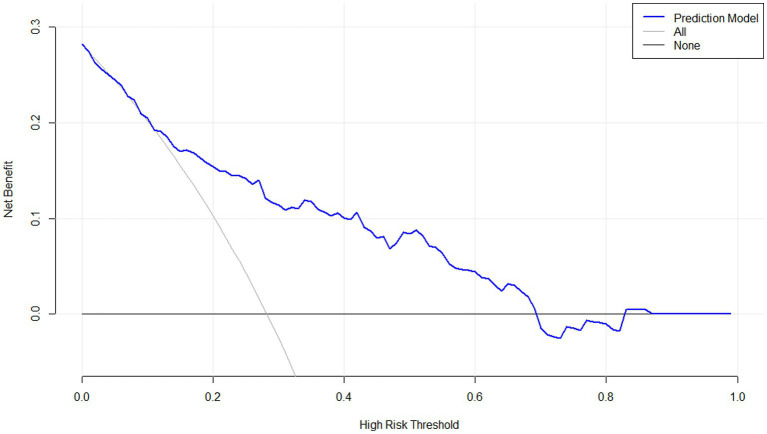
Decision curve confirming the clinical benefit of early neurological deterioration prediction model containing serum neuritin levels at admission among patients with intracerebral hemorrhage. Decision curve analysis demonstrated that such a prediction model combining serum neuritin levels at admission, Glasgow coma scale scores, and hematoma volume, relatively speaking, had a high clinical benefit.

As listed in Table 7, significantly lower GCS scores, substantially higher hematoma volumes, blood glucose levels, and serum neuritin levels, and markedly higher proportions of intraventricular hemorrhage and diabetes mellitus were confirmed in patients with END than in those without (all *p* < 0.05). Under application of the multivariate logistic regression analysis, GCS scores, hematoma volume, and serum neuritin levels were three independent predictors of END after ICH (all *p* < 0.05; Table 8). Moreover, Figure 7 shows that its discriminatory ability was similar to those of GCS scores (AUC, 0.836; 95% CI, 0.778–0.884; *p* = 0.314) and hematoma volume (AUC, 0.818; 95% CI, 0.757–0.868; *p* = 0.543). The three predictors were consolidated to establish a model, and subsequently, a nomogram was drawn for a visual manifestation of the prediction model (Figure 8). Under calibration curve evaluation, the prediction model was marked by high stability (Figure 9). Using decision curve analysis, the prediction model was clinically valid (Figure 10).

**Table 7:** Factors associated with early neurological deterioration after intracerebral hemorrhage.

	END	Non-END	*P-*value
Gender (male/female)	34/23	86/59	0.965
Age (y)	64.6 ± 13.9	62.1 ± 13.8	0.253
Current cigarette smoking	11 (19.3%)	37 (25.5%)	0.350
Alcohol abuse	18 (31.6%)	37 (25.5%)	0.384
Hypertension	32 (56.1%)	97 (66.9%)	0.152
Diabetes mellitus	18 (31.6%)	24 (16.6%)	**0.018**
Hyperlipidemia	12 (21.1%)	27 (18.6%)	0.693
Use of statins drugs	10 (17.5%)	15 (10.3%)	0.162
Use of antiplatelet drugs	9 (15.8%)	20 (13.8%)	0.716
Use of anticoagulation drugs	4 (7.0%)	11 (7.6%)	0.890
Hospital admission time (h)	5.0 (3.0–7.0)	6.0 (4.0–8.0)	0.091
Blood-sampling time (h)	5.5 (4.0–8.5)	7.0 (5.0–9.0)	0.071
Lobar hemorrhage	15 (26.3%)	30 (20.7%)	0.387
Infratentorial hemorrhage	7 (12.3%)	22 (15.2%)	0.598
Intraventricular hemorrhage	15 (26.3%)	15 (10.3%)	**0.004**
Subarachnoid hemorrhage	7 (12.3%)	6 (4.1%)	0.052
GCS scores	10 (9–11)	13 (12–14)	**<0.001**
Hematoma volume (ml)	30.1 (19.2–40.2)	12.8 (7.2–18.9)	**<0.001**
Systolic arterial pressure (mmHg)	154.6 ± 24.0	153.9 ± 21.0	0.846
Diastolic arterial pressure (mmHg)	91.7 ± 16.6	88.9 ± 14.7	0.241
Blood glucose levels (mmol/l)	7.4 (6.1–9.7)	6.6 (5.1–8.3)	**0.013**
Blood potassium levels (mmol/l)	3.7 (3.5–4.0)	3.7 (3.5–3.9)	0.456
Blood leucocyte count (×10^9^/l)	7.6 (6.0–10.2)	7.6 (5.9–9.6)	0.610
Blood C-reactive protein levels (mg/l)	12.8 (5.8–17.6)	11.0 (5.2–15.0)	0.240
Blood neuritin levels (pg/ml)	249.3 (220.3–268.7)	194.3 (170.3–227.9)	**<0.001**

Data were shown as mean ± standard deviation, median (25th–75th percentiles) or count (percentage) where appropriate. Statistical methods for intergroup comparison included the *t-*test, Mann–Whitney *U* test, Pearson chi-square test or Fisher’s exact test as appropriate. GCS means Glasgow coma scale; END, early neurological deterioration. Bold values indicate *p* < 0.05.

**Table 8:** Multivariate logistic regression analysis for risk factors of early neurological deterioration in intracerebral hemorrhage.

	Odds ratio (95% confidence interval)	*P-*value
Diabetes mellitus	2.173 (0.784–6.026)	0.136
Intraventricular hemorrhage	1.454 (0.425–4.982)	0.551
GCS scores	0.636 (0.509–0.795)	**0.001**
Hematoma volume (ml)	1.069 (1.025–1.115)	**0.002**
Blood glucose levels (mmol/l)	1.036 (0.898–1.194)	0.631
Blood neuritin levels (pg/ml)	1.013 (1.002–1.025)	**0.024**

Results were showed as odds ratio (95% confidence interval) according to the binary logistic regression analysis. Bold values indicate *p* < 0.05.

The combined model demonstrated higher END predictive efficiency for END compared to its individual components (all *p* < 0.05; Figure 7). Furthermore, no significant differences were observed in the predictive ability of both serum neuritin levels and the combined model for anticipating END under the ROC curve between all 202 patients and the subset of 101 patients (both *p* > 0.05; Supplementary Figures S12, S13).

We categorized GCS as follows: scores of 3–8 were defined as 2, scores of 9–12 as 1, and scores of 13–15 as 0. Hematoma volume was categorized as ≥30 mL (defined as 1) < 30 mL (defined as 0). Serum neuritin levels were categorized as ≥242.5 pg./mL (defined as 1) and <242.5 pg./mL (defined as 0). The percentages of patients with END increased progressively, with total scores ranging from 0 to 4: 6.5% (5/77), 12.3% (8/65), 56.3% (18/32), 94.7% (18/19), and 88.9% (8/9), respectively.

## 4 Discussion

To the best of our knowledge, there have been no studies on changes in serum neuritin levels in ICH patients and their relationship to prognosis and END after ICH. In the present study, we demonstrated that (1) serum neuritin levels in patients increased at admission, peaked at day 3, and then gradually decreased and were significantly higher during the 10 days after ICH than in healthy controls; (2) admission serum neuritin levels were independently associated with END and adverse outcomes at 90 days after ICH; (3) admission serum neuritin levels exhibited a similar prognostic and END predictive power as compared to GCS scores and hematoma volume; (4) the prediction models with the integration of GCS, hematoma volume and serum neuritin performed satisfactorily by using many statistical methods and internal validation. Therefore, serum neuritin may be a potential biomarker for assessing disease severity and predicting adverse outcomes as well as END in ICH patients.

Neuritin is a novel neurotrophic factor with anti-inflammatory, anti-apoptotic, and neuroprotective effects (11). A growing body of data has confirmed the protective effects of neuritin in acute brain injury. *In vitro* and *in vivo* experiments, neuritin is coordinately expressed with integrin, myelin oligodendrocyte glycoprotein, and microtubule-associated protein 1A and collaboratively promotes neuronal regeneration and inhibits neuronal apoptosis (21–24). In the ICH mouse model, a large amount of neuritin was expressed in neurons, significantly reducing brain injury, brain edema, and neuronal apoptosis caused by ICH (13). Similarly, in the subarachnoid hemorrhage and traumatic brain injury experiment, neuritin can significantly reduce the destruction of the blood–brain barrier, brain edema, and cell apoptosis in rats, thus playing a neuroprotective role (16, 25). Another experimental study showed that neuritin-overexpressing transgenic mice demonstrate enhanced neuroregenerative capacity and that neuritin could improve outcomes following cerebral ischemia injury (26). Thus, neuritin could play a neuroprotective role in acute brain injury, and subsequently, neuritin may become a therapeutic target of acute brain injury.

Neuritin is expressed in many tissues, but it is mainly concentrated in the neuronal soma (11, 21). Neuritin showed a persistent activation in the frontal-cingulate cortex in a rat model of cerebral ischemia (27). After acute brain injury, the expression of neuritin in the brain tissues of rats with subarachnoid hemorrhage and mice with cerebral ischemia was significantly upregulated (15, 26). Hence, considering the neuroprotective effect of neuritin, we speculate that the increased expression of neuritin may be a compensatory response to acute brain injury.

One study confirmed that neuritin can be released in brain tissue after acute brain injury (13). Therefore, neuritin may leak into the peripheral blood through the damaged blood–brain barrier, resulting in increased neuritin levels in the peripheral blood. In this study, there were no significant differences in baseline features between all 202 patients and those 54 consenting for blood collections at multiple time points after ICH, indicating that those 54 patients may be representative of the whole patients from a statistical perspective. Our study further found that serum neuritin levels were significantly higher in ICH patients compared to healthy controls, with the highest value on day 3 post-ICH. It is concluded that the neuritin in the serum of ICH patients may be at least partially derived from the injured brain tissue.

To the best of our knowledge, it is unclear whether serum neuritin is strongly associated with poor long-term outcomes in ICH patients. Neuritin has anti-inflammatory, anti-apoptotic, and neuroprotective effects (11). Hence, we speculate that serum neuritin may be a predictor of 90-day post-ICH adverse outcomes. In this study, adverse outcomes were defined as mRS scores ≥3. Final results showed that serum neuritin levels, besides GCS scores and hematoma volume, were independently associated with poor prognosis. In addition, serum neuritin levels, GCS scores, and hematoma volume had similar prognostic abilities under the ROC curve. Our findings provide further evidence to support the hypothesis that serum neuritin may be a useful biomarker for assessing disease severity after ICH.

It is well known that GCS score and hematoma volume are commonly used clinically to evaluate the poor prognosis of ICH patients (28, 29). In this study, our results suggest that serum neuritin was also a prognostic determinant of ICH. In addition, serum neuritin levels were independently associated with END, which occurs after ICH, leading to a poorer prognosis in such patients. The results showed that serum neuritin levels, compared with GCS score and hematoma volume, had a certain value in predicting the prognosis of aSAH. These results may strongly support the hypothesis that serum neuritin may represent a promising prognostic biomarker for ICH. However, at present, the known studies of other researchers only included ICH animal model experiments, and there are no relevant studies on the relationship between peripheral blood neuritin and acute brain injury in humans. This is probably a preliminary study on the role of serum neuritin as a prognostic biomarker for ICH. Larger cohort studies are needed in the future to verify this conclusion.

In our study, an interesting finding is that the prediction models with the integration of GCS, serum neuritin, and hematoma volume performed well in the prediction of poor prognosis and END. The models’ stability and clinical validity have been verified using a calibration curve and decision curve. In the current study, the restricted cubic splines have been plotted, which confirmed the linear relations existed between serum neuritin levels and the risk of poor prognosis and END. Statistically, such a result is strongly supportive of the notion that serum neuritin could be considered a continuous variable that can be integrated into the prediction models. Moreover, by using multivariate analysis, serum neuritin levels were independently correlated with GCS scores and hematoma volumes, and the levels had independent associations with ordinal mRS scores at the 90-day mark after ICH. Moreover, the models were verified for their discrimination efficiency by extracting a portion of patients. For the sake of convenience in clinical application, two exclusive weighted scaling systems were configured for anticipation of END and poor prognosis post-ICH. Such results have solidified serum neuritin as a potential biomarker for reflecting severity and forecasting poor prognosis after ICH.

There are several advantages and limitations. The advantages are that (1) as far as we are aware, this may be the first series to explore the prognostic role of serum neuritin in ICH, and therefore serum neuritin was verified to be a potential prognostic biomarker of ICH; and (2) all severity correlations and prognosis associations were demonstrated using multiple factorial analyses herein, so the conclusions may be more rational and scientific. The limitations are that (1) because a medium sample size of 202 patients was included in this study, an internal validation of the model was done only via extracting half of the entire number; (2) all conclusions were based on a medium number of patients and therefore should be validated in a larger cohort study in the future; (3) it may be of clinical value that the follow-up period is extended to 6 months and even 1 year; and (4) baseline data were incomplete in the controls.

## 5 Conclusion

To the best of our knowledge, this is the first study to quantitatively measure serum neuritin levels in ICH patients. Our study found that (1) disease severity, as reflected by GCS score and hematoma volume, is strongly correlated with serum neuritin levels, and (2) serum neuritin levels independently predict poor prognosis and END after ICH. Therefore, we speculate that neuritin may be a potential biomarker for ICH prognosis.

## Data Availability

The raw data supporting the conclusions of this article will be made available by the authors, without undue reservation.

## Footnotes

^1^ https://www.r-project.org

## Supplementary material

Study-plan diagram encompassing study-design types and participant selections. This was an observational study, containing two sub-studies, namely, the cross-sectional assessment and the prospective cohort investigation, in order to determine evolutional trajectory of serum neuritin levels and the role of serum neuritin as a prognostic biochemical marker of intracerebral hemorrhage. ICH stands for intracerebral hemorrhage.

SUPPLEMENTARY FIGURE S2 Bland-Altman plot assessing the consistency of two-measurement values of serum neuritin levels after intracerebral hemorrhage. All values of serum neuritin levels were almost located within 95% confidence interval. SD denotes standard deviation.

SUPPLEMENTARY FIGURE S3 Dynamic change of serum neuritin levels after acute intracerebral hemorrhage. Relative to healthy controls, serum neuritin levels of patients increased at admission, peaked at day 3, and then gradually decreased and were significantly higher during ten days than those of healthy controls (*P* < 0.001).

SUPPLEMENTARY FIGURE S4 Relationship between serum neuritin levels, Glasgow coma scale scores and hematoma volume among patients with intracerebral hemorrhage. **(A)** Correlative analysis of serum neuritin levels with Glasgow coma scale scores after intracerebral hemorrhage. Serum neuritin levels were significantly decreased with increased Glasgow coma scale scores using Spearman correlation coefficients (*P* < 0.001). **(B)** Correlative analysis of serum neuritin levels with hematoma volume after intracerebral hemorrhage. Serum neuritin levels were significantly correlated with hematoma volume using Spearman correlation coefficients (*P* < 0.001). **(C)** Differences in serum neuritin levels among subgroups with different Glasgow coma scale scores following intracerebral hemorrhage. There were statistical differences in serum neuritin levels among subgroups with different Glasgow coma scale scores, with highest levels in patients with Glasgow coma scale scores 3–8 and lowest levels in those with Glasgow coma scale scores 13–15 using Kruskal–Wallis *H*-test (*P* < 0.001). **(D)** Differences in serum neuritin levels between two groups across hematoma volume. Patients with hematoma volume above 30 ml had substantially higher serum neuritin levels than those with hematoma volume below 30 ml using Mann–Whitney *U*-test (*P* < 0.001). GCS signifies Glasgow Coma Scale.

SUPPLEMENTARY FIGURE S5 Difference of serum neuritin levels in intracerebral hemorrhage patients with good outcome and poor outcome. Using Mann–Whitney *U*-test, serum neuritin levels were significantly higher in patients with poor outcome than in patients with good outcome (*P* < 0.001).

SUPPLEMENTARY FIGURE S6 Relationship between serum neuritin levels and modified Rankin Scale scores at 90 days after intracerebral hemorrhage. **(A)** Bivariate correlation between serum neuritin levels and modified Rankin Scale scores at 90-day mark following intracerebral hemorrhage. Serum neuritin levels were substantially, positively correlated with modified Rankin Scale scores using Spearman correlation coefficients (*P* < 0.001). **(B)** Serum neuritin levels among subgroups with different modified Rankin Scale scores post-acute intracerebral hemorrhage. Statistical differences existed in serum neuritin levels among subgroups with different modified Rankin Scale scores, with highest levels in patients with modified Rankin Scale score 5 and lowest levels in those with modified Rankin Scale score 0 using Kruskal–Wallis *H*-test (*P* < 0.001). mRS indicates modified Rankin Scale.

SUPPLEMENTARY FIGURE S7 Restricted cubic spline of serum neuritin levels for risk of poor prognosis at 90 days after intracerebral hemorrhage. The curve showed linear dose-response associations of serum neuritin levels with risk of poor prognosis (*P* for non-linear > 0.05).

SUPPLEMENTARY FIGURE S8 Receiver operating characteristic curve assessing prognostic predictive ability of serum neuritin levels between all patients and randomly extracted patients. Serum neuritin levels possessed similar prognostic predictive ability between all patients and randomly extracted patients (*P* > 0.05). AUC denotes area under curve; 95% CI, 95% confidence interval; ns, non-significant.

SUPPLEMENTARY FIGURE S9 Receiver operating characteristic curve assessing prognostic predictive ability of combined model between all patients and randomly extracted patients. Combined model possessed similar prognostic predictive ability between all patients and randomly extracted patients (*P* > 0.05). AUC denotes area under curve; 95% CI, 95% confidence interval; GCS, Glasgow coma scale; ns, non-significant.

SUPPLEMENTARY FIGURE S10 Difference of serum neuritin levels between intracerebral hemorrhage patients with early neurological deterioration and those without. Using Mann–Whitney *U*-test, serum neuritin levels were significantly higher in patients with early neurological deterioration than in patients without that (*P* < 0.001). END indicates early neurological deterioration.

SUPPLEMENTARY FIGURE S11 Restricted cubic spline of serum neuritin levels for risk of early neurological deterioration after intracerebral hemorrhage. The curve showed linear dose-response associations of serum neuritin levels with risk of early neurological deterioration (*P* for non-linear > 0.05). END indicates early neurological deterioration.

SUPPLEMENTARY FIGURE S12 Receiver operating characteristic curve assessing early neurological deterioration predictive ability of serum neuritin levels between all patients and randomly extracted patients. Serum neuritin levels possessed similar early neurological deterioration predictive ability between all patients and randomly extracted patients (*P* > 0.05). AUC denotes area under curve; 95% CI, 95% confidence interval; ns, non-significant.

SUPPLEMENTARY FIGURE S13 Receiver operating characteristic curve assessing early neurological deterioration predictive ability of combined model between all patients and randomly extracted patients. Combined model possessed similar early neurological deterioration predictive ability between all patients and randomly extracted patients (*P* > 0.05). AUC denotes area under curve; 95% CI, 95% confidence interval; GCS, Glasgow coma scale; ns, non-significant.
