# Supplementary material for: Serum neuritin as a predictive biomarker of early neurological deterioration and poor prognosis after spontaneous intracerebral hemorrhage: a prospective cohort study
Source: Front Neurol. 2025 Jan 7;15:1490023. doi: 10.3389/fneur.2024.1490023 (PMC11746067; doi:10.3389/fneur.2024.1490023)
Supplement: Supplementary file 1 [file Table_1.doc]

**Supplemental Table 1** Differences in baseline parameters between all patients and other two groups of patients with intracerebral hemorrhage

|  | All 202 patients | Partial 54 patients | Partial 101 patients | P1 value | P2 value |
| --- | --- | --- | --- | --- | --- |
| Gender (male/female) | 120/82 | 35/19 | 64/37 | 0.470 | 0.506 |
| Age (y) | 62.8±13.8 | 62.5±12.9 | 62.2±12.8 | 0.866 | 0.716 |
| Current cigarette smoking | 48 (23.8%) | 12 (22.2%) | 23 (22.8%) | 0.812 | 0.848 |
| Alcohol abuse | 55 (27.2%) | 19 (35.2%) | 23 (22.8%) | 0.252 | 0.403 |
| Hypertension | 129 (63.9%) | 30 (55.6%) | 57 (56.4%) | 0.264 | 0.211 |
| Diabetes mellitus | 42 (20.8%) | 16 (29.6%) | 26 (25.7%) | 0.168 | 0.330 |
| Hyperlipidemia | 39 (19.3%) | 12 (22.2%) | 21 (20.8%) | 0.634 | 0.760 |
| Use of statins drugs | 25 (12.4%) | 6 (11.1%) | 11 (10.9%) | 0.800 | 0.706 |
| Use of antiplatelet drugs | 29 (14.4%) | 10 (18.5%) | 15 (14.9%) | 0.450 | 0.908 |
| Use of anticoagulation drugs | 15 (7.4%) | 3 (5.6%) | 5 (5.0%) | 0.633 | 0.413 |
| Hospital admission time (h) | 5.9 (4.0-8.0) | 5.0 (3.0-7.5) | 6.0 (4.0-8.0) | 0.174 | 0.604 |
| Blood-sampling time (h) | 6.0 (4.5-9.0) | 5.8 (4.0-8.5) | 6.5 (5.0-9.0) | 0.169 | 0.576 |
| Lobar hemorrhage | 45 (22.3%) | 11 (20.4%) | 28 (27.7%) | 0.763 | 0.296 |
| Infratentorial hemorrhage | 29 (14.4%) | 5 (9.3%) | 14 (13.9%) | 0.327 | 0.907 |
| Intraventricular hemorrhage | 30 (14.9%) | 11 (20.4%) | 19 (18.8%) | 0.326 | 0.377 |
| Subarachnoid hemorrhage | 13 (6.4%) | 4 (7.4%) | 8 (7.9%) | 0.799 | 0.631 |
| Glasgow coma scale scores | 12 (10-14) | 12 (10-13) | 12 (10-14) | 0.180 | 0.150 |
| Hematoma volume (ml) | 15.3 (8.6-25.5) | 16.9 (10.6-28.2) | 15.9 (8.7-28.2) | 0.326 | 0.660 |
| Systolic arterial pressure (mmHg) | 154.1±21.8 | 154.8±22.9 | 150.0±22.8 | 0.825 | 0.129 |
| Diastolic arterial pressure (mmHg) | 89.7±15.3 | 89.7±14.9 | 88.3±15.7 | 0.983 | 0.468 |

Data were shown as mean ± standard deviation, median (25th-75th percentiles) or count (percentage) as appropriate. Statistical methods encompassed the t test, Mann–Whitney U test, Pearson chi-square test or Fisher's exact test as appropriate. In this study, a total of 202 patients were enrolled, 54 patients agreed with blood drawings at numerous time points after stroke and 101 patients were randomly extracted for internal validation of combination model. P1 values were generated from comparison of baseline data between all patients and 54 patients; and P2 values were yielded from comparison of baseline data between all patients and 101 patients.
