# Supplementary figures and images for: Serum neuritin as a predictive biomarker of early neurological deterioration and poor prognosis after spontaneous intracerebral hemorrhage: a prospective cohort study
Source: Front Neurol. 2025 Jan 7;15:1490023. doi: 10.3389/fneur.2024.1490023 (PMC11746067; doi:10.3389/fneur.2024.1490023)

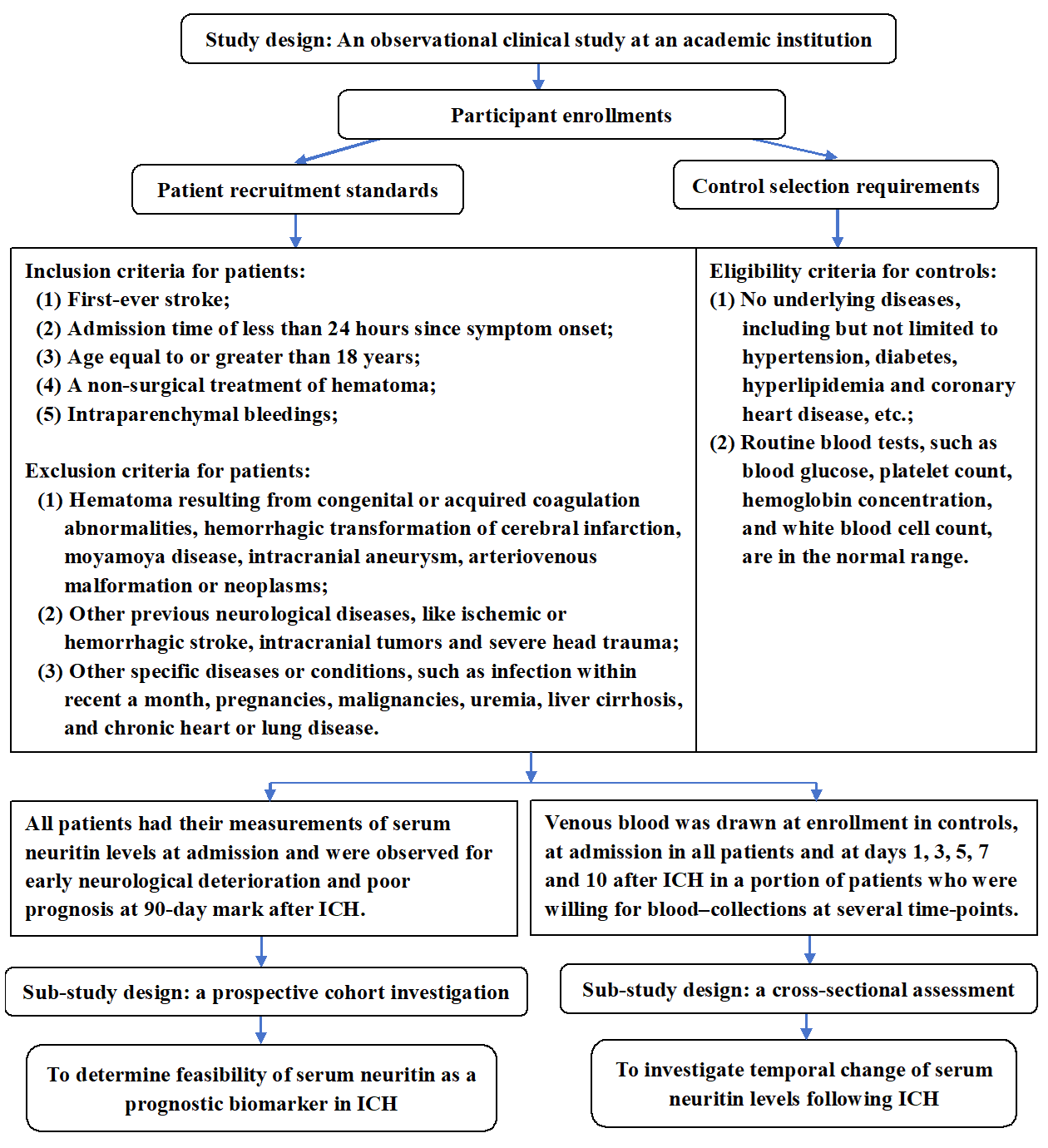

Supplement: Supplementary file 2 — Study-plan diagram encompassing study-design types and participant selections. This was an observational study, containing two sub-studies, namely, the cross-sectional assessment and the prospective cohort investigation, in order to determine evolutional trajectory of serum neuritin levels and the role of serum neuritin as a prognostic biochemical marker of intracerebral hemorrhage. ICH stands for intracerebral hemorrhage. [file Image_1.TIF]

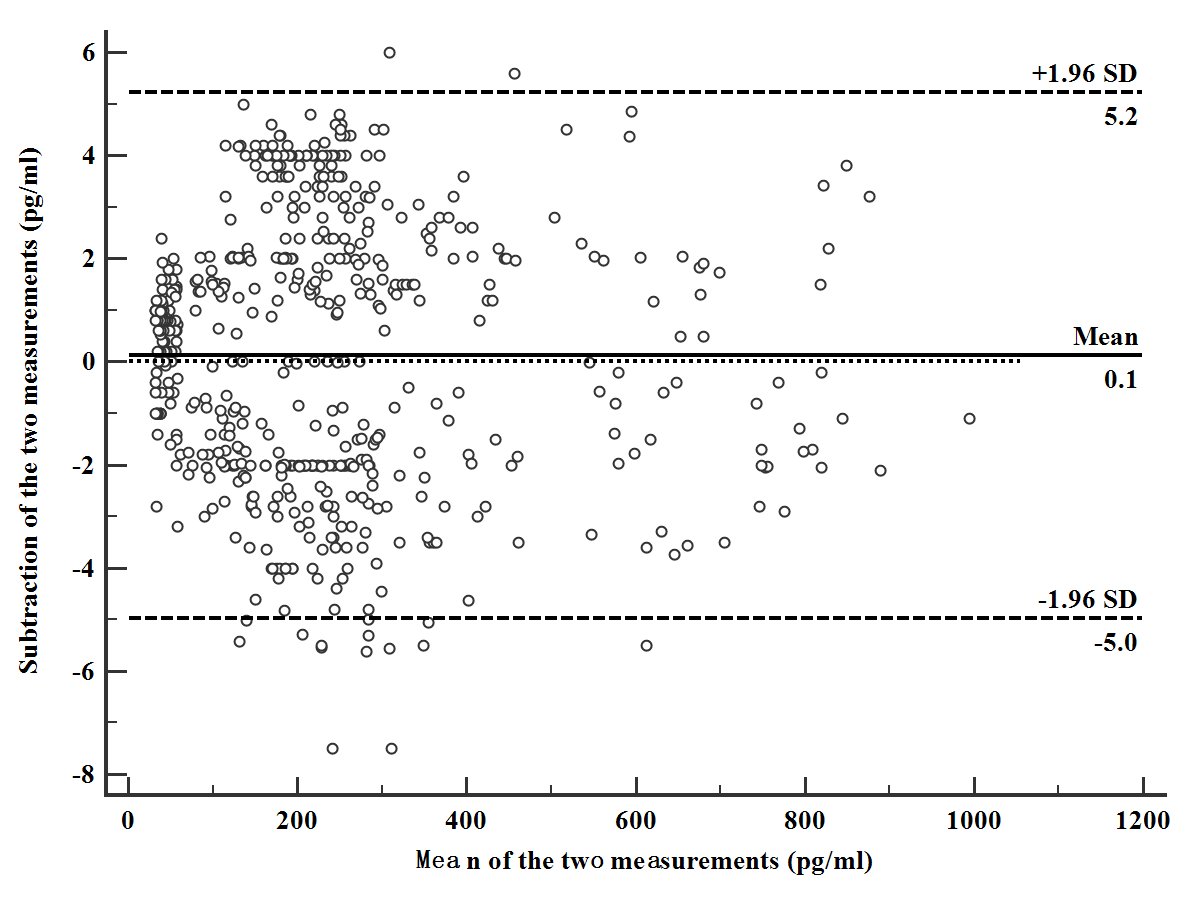

Supplement: SUPPLEMENTARY FIGURE S2 — Bland-Altman plot assessing the consistency of two-measurement values of serum neuritin levels after intracerebral hemorrhage. All values of serum neuritin levels were almost located within 95% confidence interval. SD denotes standard deviation. [file Image_2.TIF]

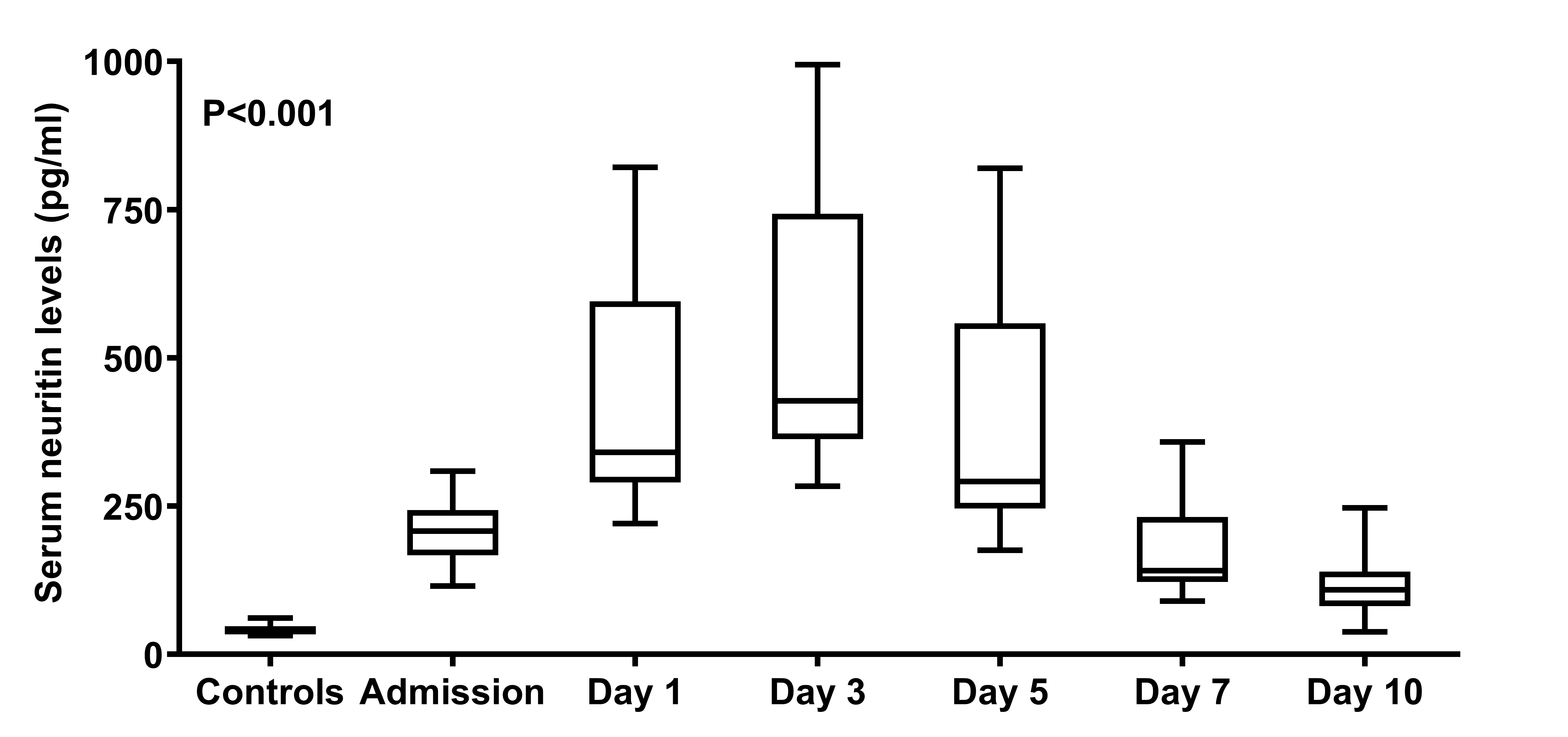

Supplement: SUPPLEMENTARY FIGURE S3 — Dynamic change of serum neuritin levels after acute intracerebral hemorrhage. Relative to healthy controls, serum neuritin levels of patients increased at admission, peaked at day 3, and then gradually decreased and were significantly higher during ten days than those of healthy controls (P < 0.001). [file Image_3.TIF]

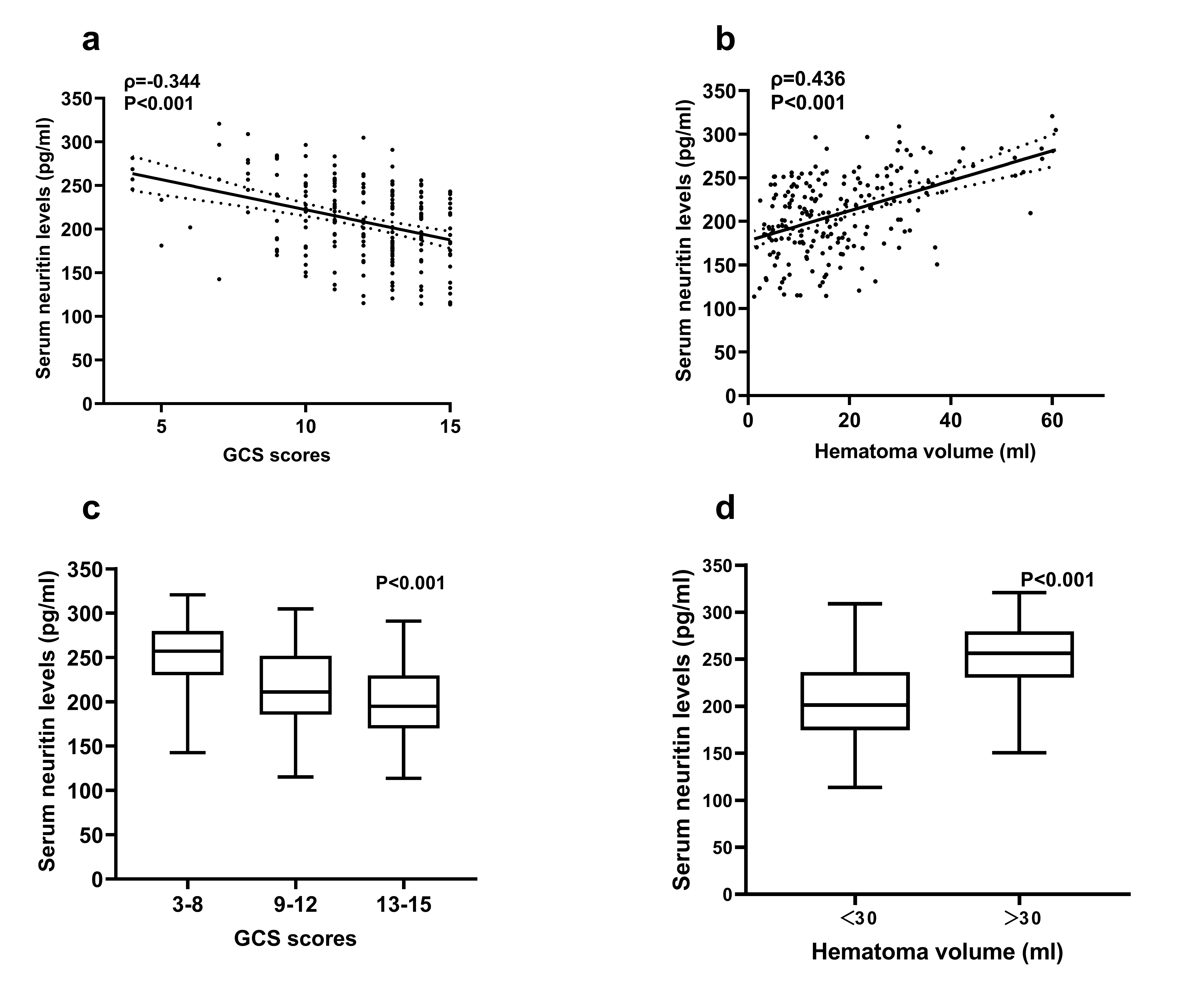

Supplement: SUPPLEMENTARY FIGURE S4 — Relationship between serum neuritin levels, Glasgow coma scale scores and hematoma volume among patients with intracerebral hemorrhage. (A) Correlative analysis of serum neuritin levels with Glasgow coma scale scores after intracerebral hemorrhage. Serum neuritin levels were significantly decreased with increased Glasgow coma scale scores using Spearman correlation coefficients (P < 0.001). (B) Correlative analysis of serum neuritin levels with hematoma volume after intracerebral hemorrhage. Serum neuritin levels were significantly correlated with hematoma volume using Spearman correlation coefficients (P < 0.001). (C) Differences in serum neuritin levels among subgroups with different Glasgow coma scale scores following intracerebral hemorrhage. There were statistical differences in serum neuritin levels among subgroups with different Glasgow coma scale scores, with highest levels in patients with Glasgow coma scale scores 3–8 and lowest levels in those with Glasgow coma scale scores 13–15 using Kruskal–Wallis H-test (P < 0.001). (D) Differences in serum neuritin levels between two groups across hematoma volume. Patients with hematoma volume above 30 ml had substantially higher serum neuritin levels than those with hematoma volume below 30 ml using Mann–Whitney U-test (P < 0.001). GCS signifies Glasgow Coma Scale. [file Image_4.TIF]

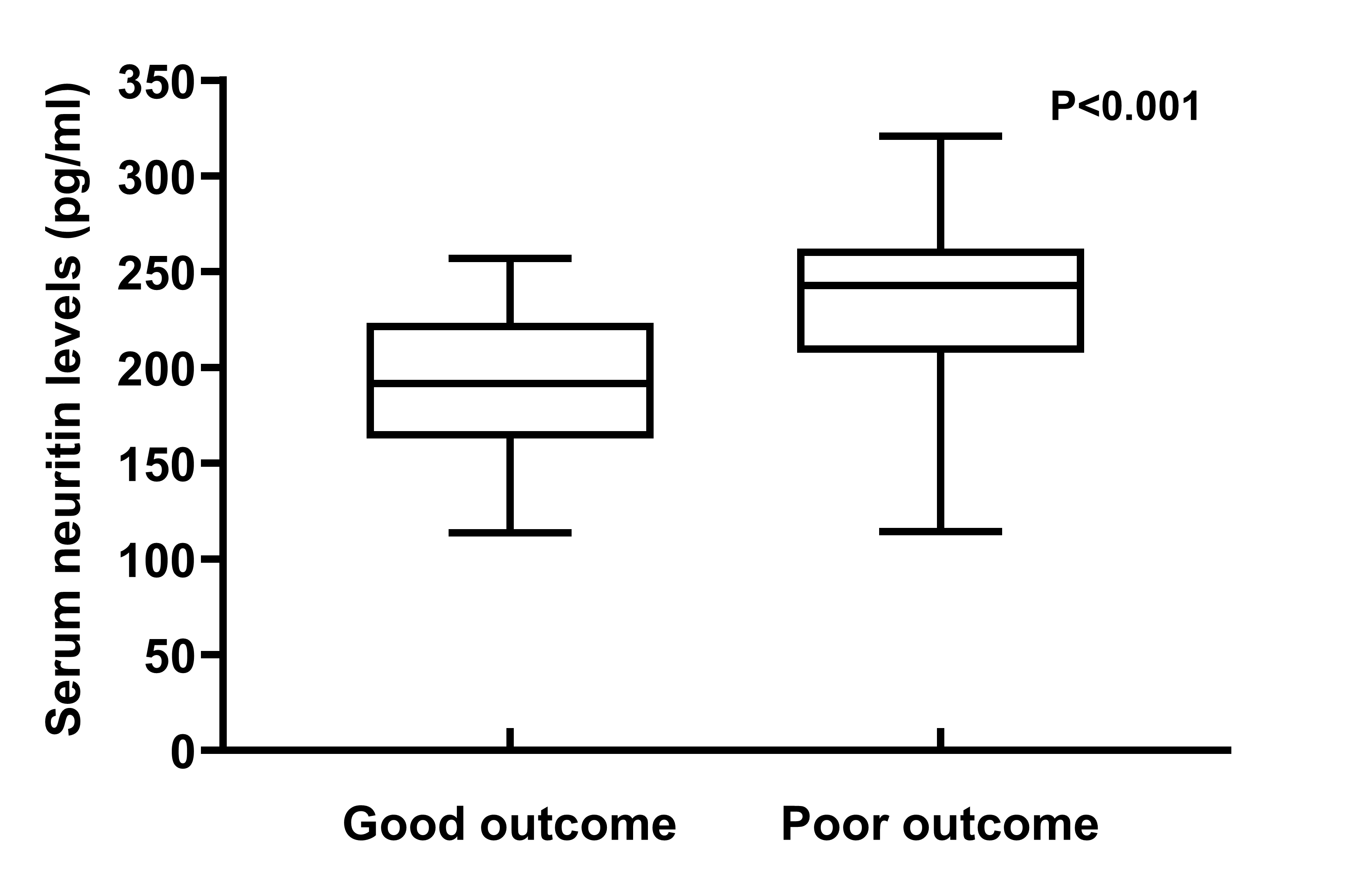

Supplement: SUPPLEMENTARY FIGURE S5 — Difference of serum neuritin levels in intracerebral hemorrhage patients with good outcome and poor outcome. Using Mann–Whitney U-test, serum neuritin levels were significantly higher in patients with poor outcome than in patients with good outcome (P < 0.001). [file Image_5.TIF]

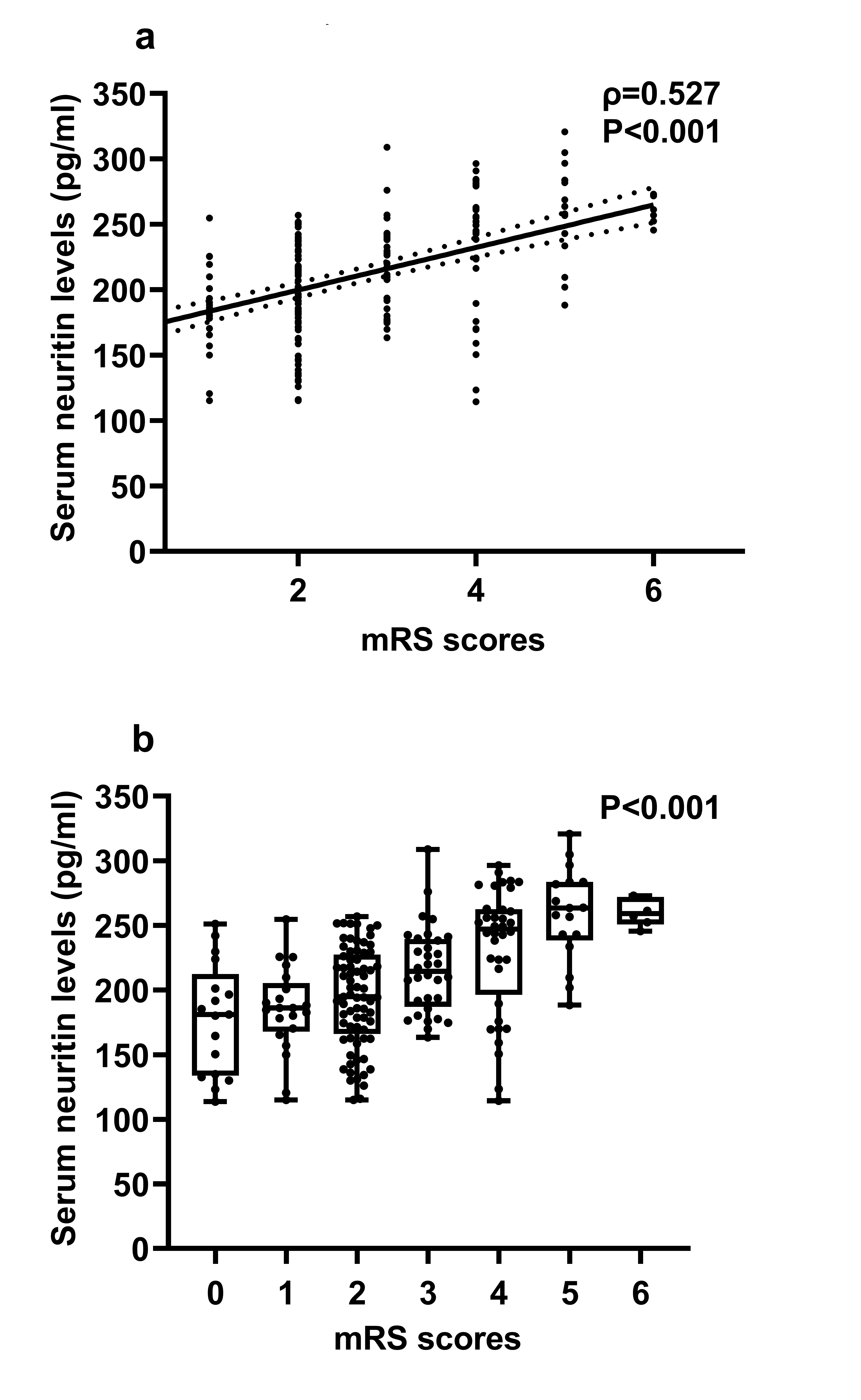

Supplement: SUPPLEMENTARY FIGURE S6 — Relationship between serum neuritin levels and modified Rankin Scale scores at 90 days after intracerebral hemorrhage. (A) Bivariate correlation between serum neuritin levels and modified Rankin Scale scores at 90-day mark following intracerebral hemorrhage. Serum neuritin levels were substantially, positively correlated with modified Rankin Scale scores using Spearman correlation coefficients (P < 0.001). (B) Serum neuritin levels among subgroups with different modified Rankin Scale scores post-acute intracerebral hemorrhage. Statistical differences existed in serum neuritin levels among subgroups with different modified Rankin Scale scores, with highest levels in patients with modified Rankin Scale score 5 and lowest levels in those with modified Rankin Scale score 0 using Kruskal–Wallis H-test (P < 0.001). mRS indicates modified Rankin Scale. [file Image_6.TIF]

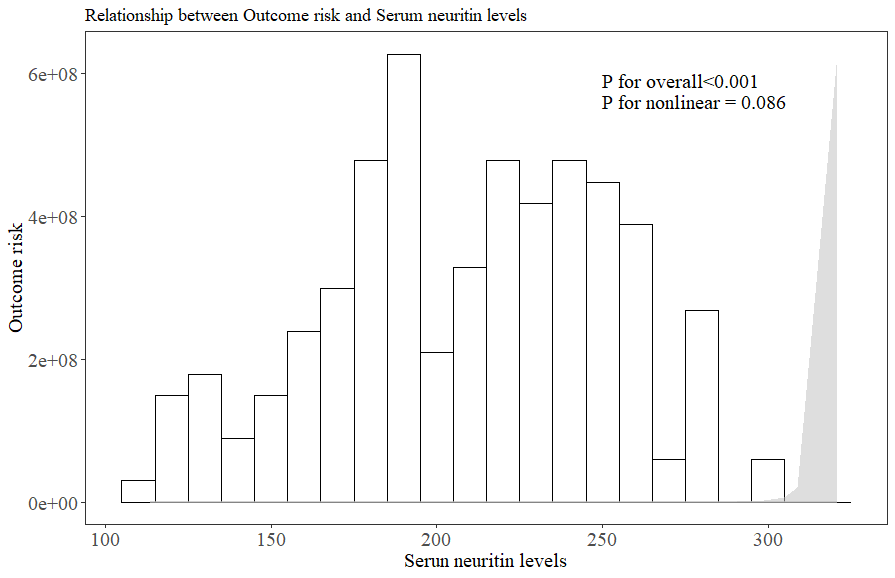

Supplement: SUPPLEMENTARY FIGURE S7 — Restricted cubic spline of serum neuritin levels for risk of poor prognosis at 90 days after intracerebral hemorrhage. The curve showed linear dose-response associations of serum neuritin levels with risk of poor prognosis (P for non-linear > 0.05). [file Image_7.TIFF]

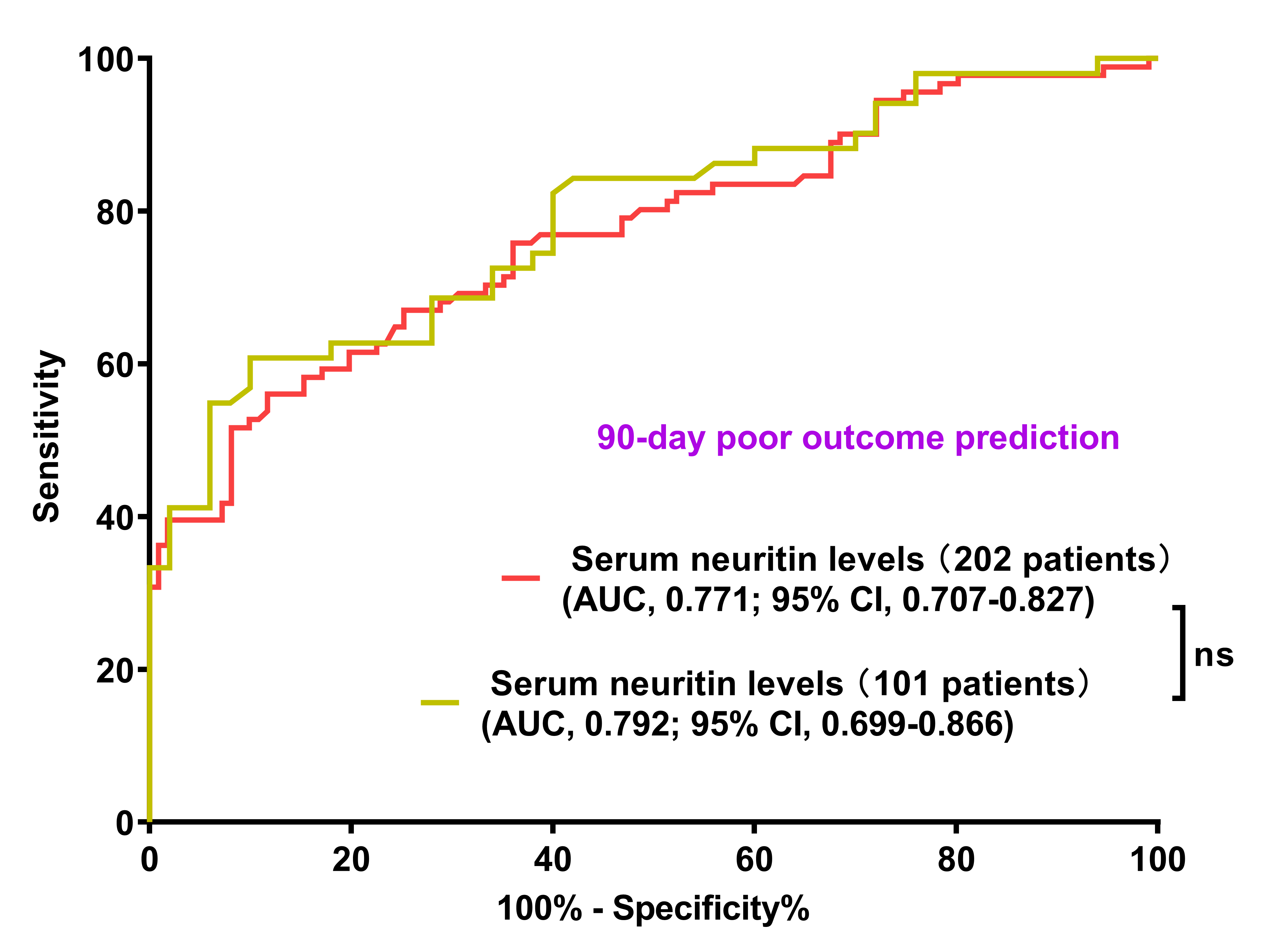

Supplement: SUPPLEMENTARY FIGURE S8 — Receiver operating characteristic curve assessing prognostic predictive ability of serum neuritin levels between all patients and randomly extracted patients. Serum neuritin levels possessed similar prognostic predictive ability between all patients and randomly extracted patients (P > 0.05). AUC denotes area under curve; 95% CI, 95% confidence interval; ns, non-significant. [file Image_8.TIF]

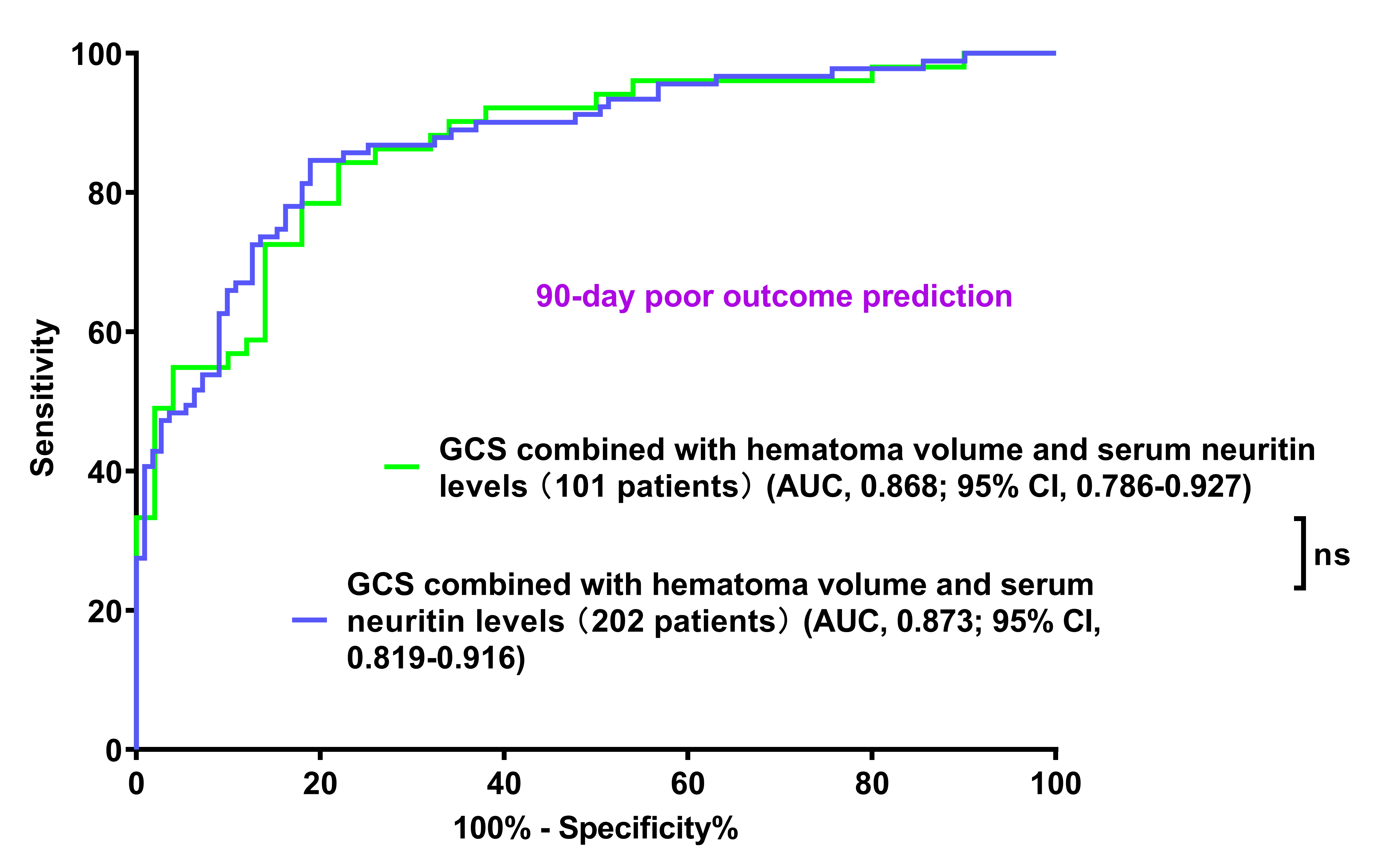

Supplement: SUPPLEMENTARY FIGURE S9 — Receiver operating characteristic curve assessing prognostic predictive ability of combined model between all patients and randomly extracted patients. Combined model possessed similar prognostic predictive ability between all patients and randomly extracted patients (P > 0.05). AUC denotes area under curve; 95% CI, 95% confidence interval; GCS, Glasgow coma scale; ns, non-significant. [file Image_9.TIF]

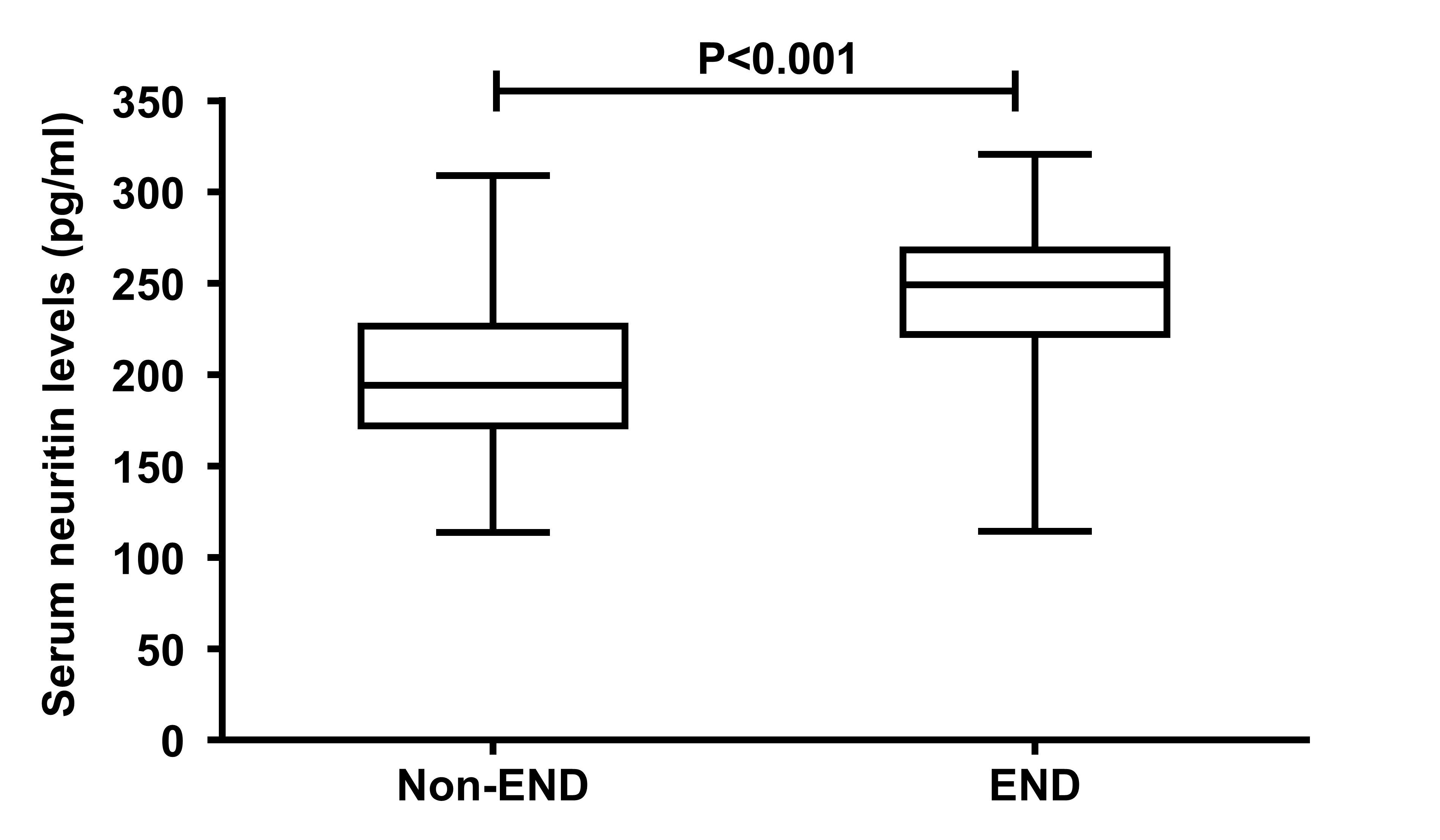

Supplement: SUPPLEMENTARY FIGURE S10 — Difference of serum neuritin levels between intracerebral hemorrhage patients with early neurological deterioration and those without. Using Mann–Whitney U-test, serum neuritin levels were significantly higher in patients with early neurological deterioration than in patients without that (P < 0.001). END indicates early neurological deterioration. [file Image_10.TIF]

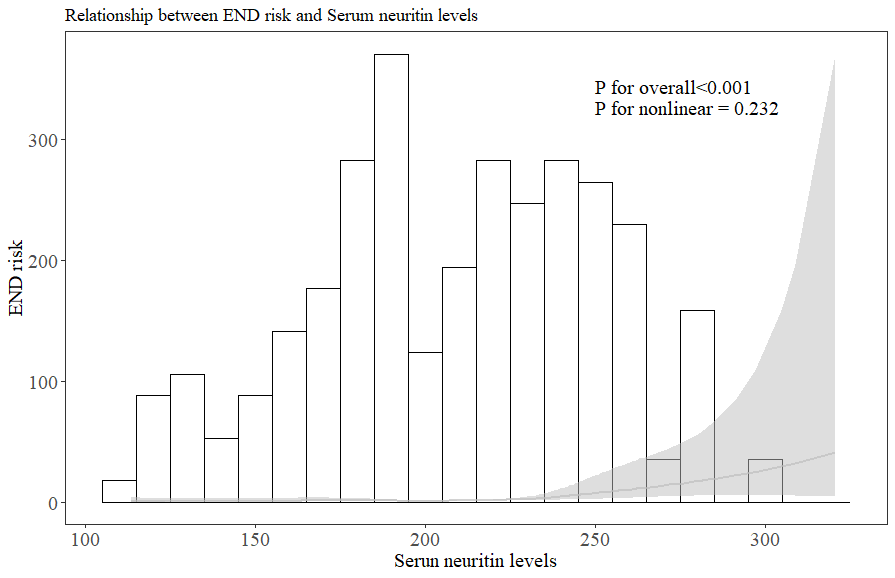

Supplement: SUPPLEMENTARY FIGURE S11 — Restricted cubic spline of serum neuritin levels for risk of early neurological deterioration after intracerebral hemorrhage. The curve showed linear dose-response associations of serum neuritin levels with risk of early neurological deterioration (P for non-linear > 0.05). END indicates early neurological deterioration. [file Image_11.TIFF]

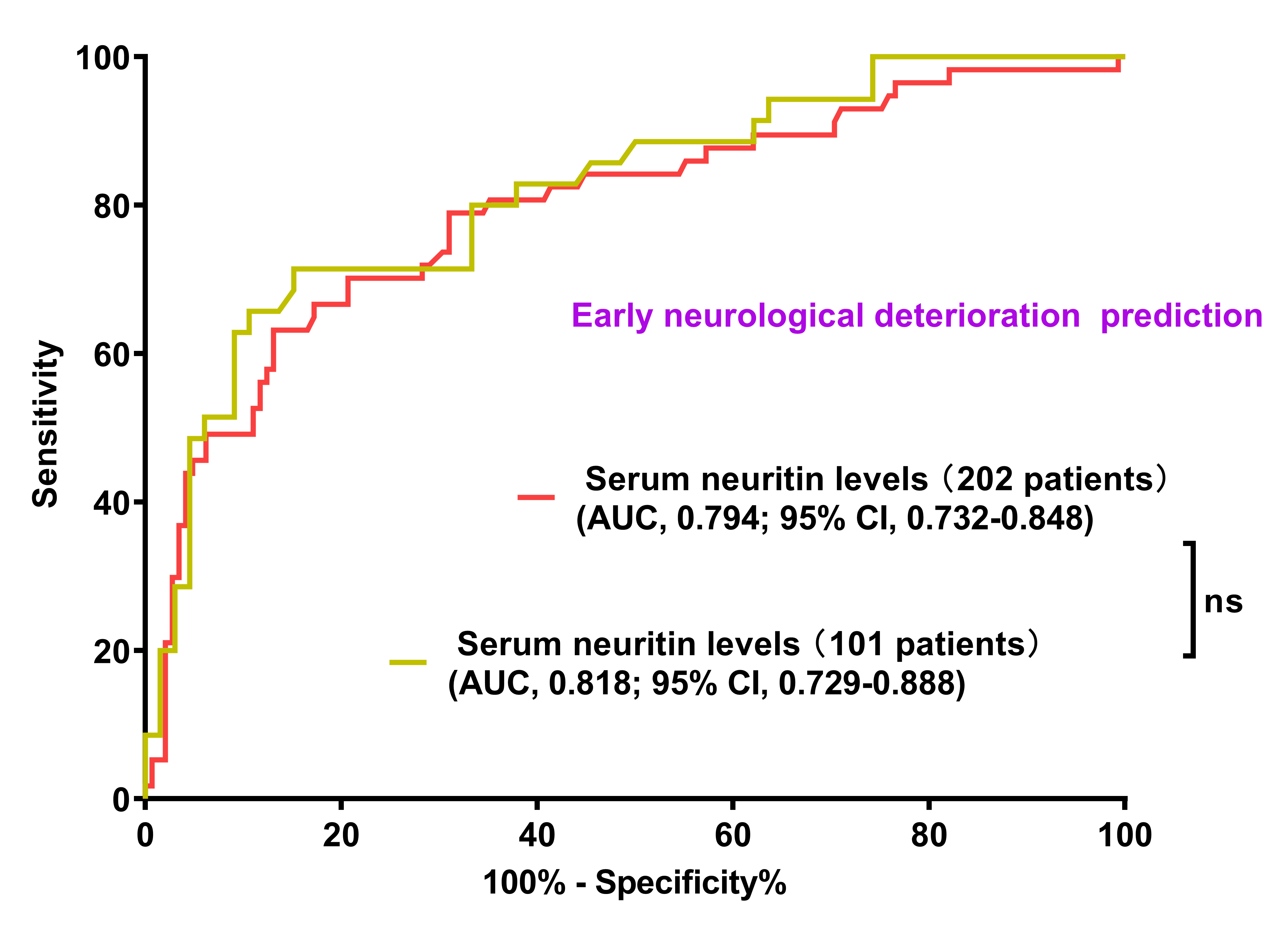

Supplement: SUPPLEMENTARY FIGURE S12 — Receiver operating characteristic curve assessing early neurological deterioration predictive ability of serum neuritin levels between all patients and randomly extracted patients. Serum neuritin levels possessed similar early neurological deterioration predictive ability between all patients and randomly extracted patients (P > 0.05). AUC denotes area under curve; 95% CI, 95% confidence interval; ns, non-significant. [file Image_12.TIF]

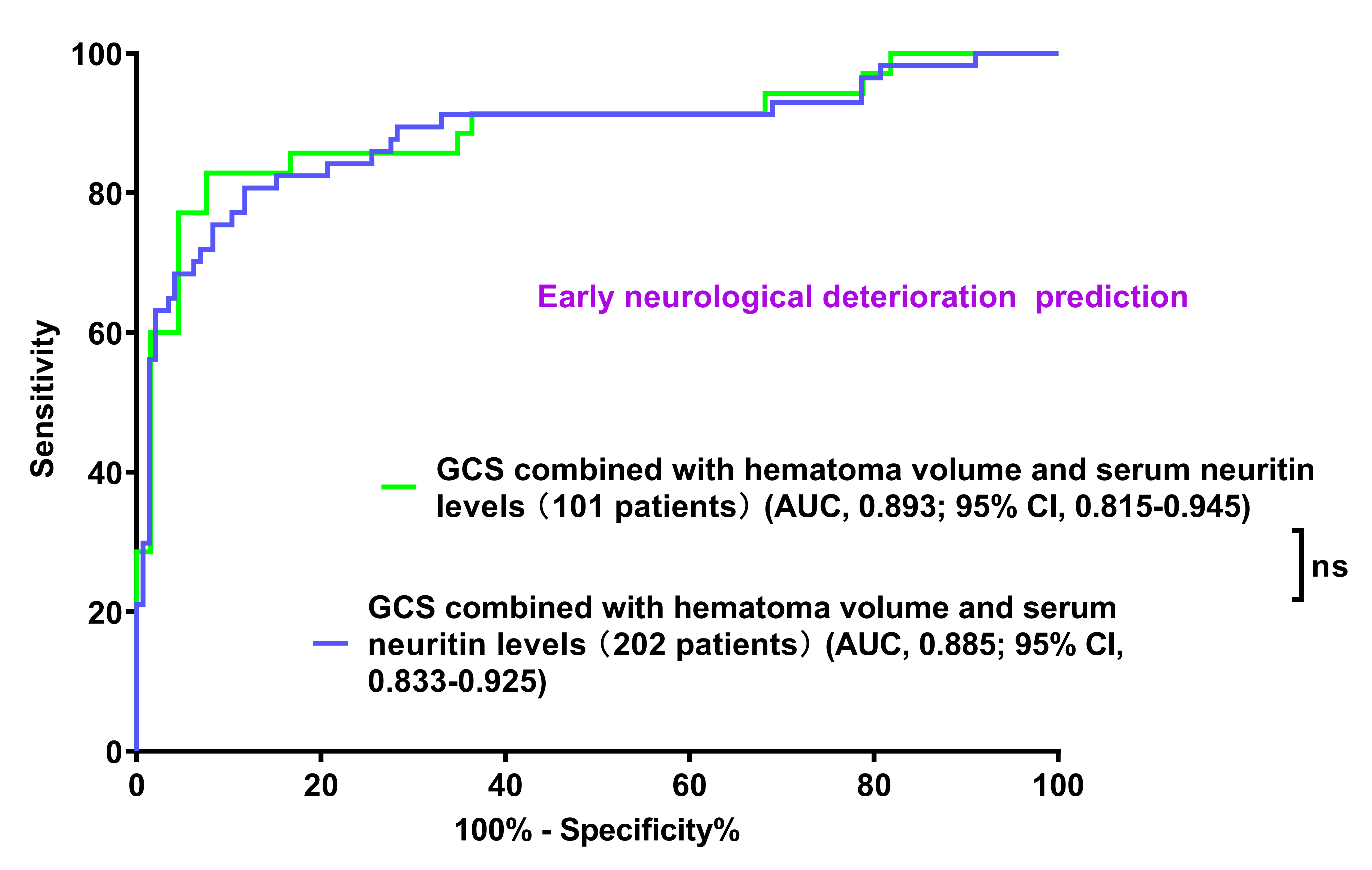

Supplement: SUPPLEMENTARY FIGURE S13 — Receiver operating characteristic curve assessing early neurological deterioration predictive ability of combined model between all patients and randomly extracted patients. Combined model possessed similar early neurological deterioration predictive ability between all patients and randomly extracted patients (P > 0.05). AUC denotes area under curve; 95% CI, 95% confidence interval; GCS, Glasgow coma scale; ns, non-significant. [file Image_13.TIF]
